# TRPM3_miR-204: a complex locus for eye development and disease

**DOI:** 10.1186/s40246-020-00258-4

**Published:** 2020-02-18

**Authors:** Alan Shiels

**Affiliations:** Ophthalmology and Visual Sciences, Washington University School of Medicine, 660 S. Euclid Ave., Box 8096, St. Louis, MO 63110 USA

**Keywords:** TRP channel, MicroRNA, Eye development, Eye disease

## Abstract

First discovered in a light-sensitive retinal mutant of *Drosophila*, the transient receptor potential (TRP) superfamily of non-selective cation channels serve as polymodal cellular sensors that participate in diverse physiological processes across the animal kingdom including the perception of light, temperature, pressure, and pain. TRPM3 belongs to the melastatin sub-family of TRP channels and has been shown to function as a spontaneous calcium channel, with permeability to other cations influenced by alternative splicing and/or non-canonical channel activity. Activators of TRPM3 channels include the neurosteroid pregnenolone sulfate, calmodulin, phosphoinositides, and heat, whereas inhibitors include certain drugs, plant-derived metabolites, and G-protein subunits. Activation of TRPM3 channels at the cell membrane elicits a signal transduction cascade of mitogen-activated kinases and stimulus response transcription factors. The mammalian TRPM3 gene hosts a non-coding microRNA gene specifying miR-204 that serves as both a tumor suppressor and a negative regulator of post-transcriptional gene expression during eye development in vertebrates. Ocular co-expression of TRPM3 and miR-204 is upregulated by the paired box 6 transcription factor (PAX6) and mutations in all three corresponding genes underlie inherited forms of eye disease in humans including early-onset cataract, retinal dystrophy, and coloboma. This review outlines the genomic and functional complexity of the TRPM3_miR-204 locus in mammalian eye development and disease.

## Background

Discovery of the transient receptor potential (TRP) cation channels traces back to visual function studies of the spontaneous, light-sensitive, retinal mutant strain (‘A-type’) of *Drosophila melanogaster* in 1969 [1–4]. Electroretinogram (ERG) recordings of these fruit fly mutants showed that their photoreceptors exhibited a transient receptor potential that decayed to baseline rather than a sustained plateau-like receptor potential characteristic of wild-type fruit flies in response to prolonged bright light. Consequently, the more aptly re-named *trp* mutant flies behaved as if ‘blinded-by-light’ due to a proposed defect in the phototransduction cascade rather than a failure of photo-pigment regeneration [2]. Eventual recombinant DNA cloning and sequencing of the *trp* ‘phototransduction’ gene in 1989, identified a large (143 kDa) type-2 transmembrane protein with 6–8 predicted transmembrane helices [5, 6] that was later found to function as a light-activated calcium ion (Ca^2+^) channel in 1992 [2–4, 7]. Simultaneously, a second light-sensitive trp-like calmodulin-binding channel (trpl) was discovered independently in *Drosophila* photoreceptors that likely accounted for the residual light response (reduced quantum bumps) observed in the *trp* mutant [3, 8]. Despite discovery of a third trp homolog (trpγ) in *Drosophila* retina [9], *trp* and *trpl* channels represent the predominant light-activated channels in *Drosophila* photoreceptors and *trp* became the founding member of the TRP ion channel superfamily [10, 11].

Based on shared sequence homology with *Drosophila trp*, the subsequent molecular cloning and sequencing of the first human ‘canonical’ TRP channel (TRPC1) in 1995 [12, 13] has led to characterization of one of the largest ion channel super-families in mammals composed of 28 members (27 in humans) that are sub-divided into six sequence-related sub-families. These are named according to structural and/or functional channel features to include, ankyrin-repeat (TRPA1), canonical or classical (TRPC1-7, human TRPC2 is a pseudogene), melastatin (TRPM1-8), vanilloid-receptor (TRPV1-6), mucolipin (TRPML1-3), and polycystin (TRPP2, 3, 5) [14–16]. While all functional TRP channels are cation permeable, they exhibit a wide range of relative divalent and monovalent cation permeabilities (e.g., Ca^2+^, Na^+^, Zn^2+^, Mg^2+^) and gating mechanisms including spontaneously active, voltage-gated, thermo-sensitive, mechanosensitive, and ligand-gated channels. Indeed, many TRP channels are polymodal responding to multiple physical and chemical stimuli and since they are widely expressed in most, if not, all mammalian cell-types (e.g., neurons, nephrons) they participate in critical physiological functions including the perception of light, temperature, pressure, and pain [17–19]. Consequently, TRP channel dysfunction has been associated with a variety of inherited and acquired diseases in humans [20–22]. So far, mutations in at least nine human TRP channel genes, across all six sub-families, have been found to underlie over a dozen genetic diseases, collectively referred to as ‘TRP channelopathies,’ that affect multiple organs including the heart (TRPM4), kidneys (TRPC6, TRPP1), skin (TRPV3), and neuro-musculoskeletal system (TRPV4) [22–26].

Among the TRP channelopathies, mutations in the human genes for two closely related members of the melastatin sub-family, TRPM1 and TRPM3, have recently been linked with inherited eye diseases. TRPM1 (melastatin-1) is the founder member of the melastatin sub-family of TRP channels (TRPM1-8) that are numbered 1–8 in order of discovery and classified into four phylogenetically conserved groups: TRPM1/3, TRPM4/5, TRPM6/7, and TRPM2/8 [27–29]. TRPM1 expression is associated with terminal differentiation of pigmented neural crest-derived cells or melanocytes, whereas loss of TRPM1 expression is a diagnostic and prognostic marker for primary cutaneous metastatic melanoma [30]. Further, expression of a non-coding microRNA–miR-211—that is located within an intron of the TRPM1 gene, is downregulated in metastatic melanoma and miR-211 has been shown to function as a tumor suppressor [30–32]. By contrast, disruption of TRPM1 expression, by genomic insertion of a retroviral long terminal repeat (LTR) sequence, has been found to cause the Leopard (*LP*) complex spotting or white (unpigmented) coat pattern of certain horse breeds (e.g., Appaloosa). Homozygous *LP* horses also suffer from a retinal disorder known as complete (no rod-photoreceptor function) congenital (present at birth) stationary (non-progressive or stable) night-blindness (nyctalopia) or cCSNB [33]. Similarly, mutation or knockout (i.e., null) of the mouse TRPM1 gene (*Trpm1*) results in a cCSNB-like phenotype [34, 35]. Accordingly, over 50 coding mutations in the human TRPM1 gene (*TRPM1*) on chromosome 15q have been shown to underlie autosomal-recessive forms of cCSNB [36]. In addition, *TRPM1* mutations (bi-allelic and deletion) can present in childhood as progressive high-myopia, involuntary eye movements (nystagmus, strabismus, or squint), and an abnormal (electronegative) full-field ERG with or without stationary or progressive night-blindness [37]. However, unlike horses, abnormal skin pigmentation has not been associated with TRPM1 deficiency or mutation in humans and mice [38, 39].

Despite its shared sequence homology with TRPM1 (~ 69% amino acid similarity, 57% amino acid identity), TRPM3 (melastatin-2) is not directly associated with melanocyte differentiation or melanoma [30]. Rather, TRPM3 channel activity in vivo has been implicated in a variety of neuronal and non-neuronal cellular processes. These include secretion of insulin by pancreatic β cells [40–42] and hyaluronic-acid from synovial fibroblasts [43], glutamatergic transmission of cerebellar Purkinje cells [44], oligodendrocyte maturation and CNS myelination [45], noxious heat-sensing by dorsal root ganglia (DRG) neurons [46–50], neuropeptide release and inflammatory pain [42], mechanosensing in vascular smooth muscle cells [51] and periodontal ligament cells during bone remodeling [52, 53], and osmosensing hypotonic stress in ductus arteriosus constriction [54] and renal epithelial cells [55]. Further, TRPM3 channel activity has been shown to promote the growth of clear cell renal cell carcinoma by stimulating oncogenic (macro) autophagy dependent on microtubule-associated protein 1 light chain 3α (LC3A) and LC3B [56].

In vitro functional expression studies have determined that TRPM3 channels are activated by several structurally unrelated agonists including (1) the endogenous excitatory neurosteroid pregnenolone sulfate, which can also weakly activate TRPM1 channels [51, 57–63]; (2) cell-membrane phosphoinositol phosphates [64–66]; and (3) noxious heat [46–50]. Conversely, they are inhibited by (1) certain clinically approved drugs including nonsteroidal anti-inflammatory drugs (NSAIDs, e.g., mefenamic acid), antibiotics (e.g., voriconazole, which also inhibits TRPM1 channels), sex-steroids (e.g., progesterone), and synthetic drug-like antagonists [40, 41, 51, 61, 67–72]; (2) naturally occurring plant-derived secondary metabolites (e.g., citrus flavanones) [73–75]; and (3) G-protein coupled receptor βγ subunits [76–79]. By contrast, TRPM1 channels were found to be inhibited by interaction with both Gα or Gβγ subunits [80].

Recent genetic studies have discovered that mutation of the human TRPM3 gene underlies an inherited form of early-onset or pediatric cataract with or without glaucoma [81, 82]. Subsequently, mutation of the human gene for microRNA-204 (miR-204) that is located within an intron of the TRPM3 gene (reminiscent of TRPM1 and miR-211) has been shown to underlie an inherited form of retinal dystrophy and ocular coloboma [83]. This gene-centric review focuses on the complex genomic organization, sequence variation, transcriptional regulation, and functional expression profile of the TRPM3_miR-204 locus within the context of eye development and disease.

## Review methodology

Comprehensive literature and bioinformatics searches were conducted via the National Center for Biotechnology Information (NCBI) website (www.ncbi.nlm.gov) through December 2019. Keyword searches of PubMed yielded approximately 200 articles for TRPM3 and over 520 articles for miR-204. Searches were refined by combination with keywords for eye tissues (e.g., cornea(l), lens, ciliary body, retina(l), optic nerve) and eye diseases (e.g., cataract, glaucoma, retinoblastoma). Bioinformatics data was obtained from several NCBI databases including Gene, Variation Viewer, and Online Mendelian Inheritance in Man (OMIM). Human and mouse bioinformatics data was derived from the Genome Reference Consortium Human Build 38 patch release 12 or 13 (GRCh38.p12/13) and the Genome Reference Consortium Mouse Build 38 patch release 6 (GRCm38.p6), respectively. NCBI searches were supported by comprehensive searches of other public bioinformatics databases including BioGPS (www.biogps.org), Ensemble Genome Browser (www.ensemble.org), Mouse Genome Informatics (MGI) (www.informatics.jax.org), and the University of California Santa Cruz (UCSC) Genome Browser (https://genome.ucsc.edu).

## TRPM3_miR-204 gene organization and variation

Both the human and mouse TRPM3 genes host a microRNA gene and exhibit extensive alternative splicing and genetic variation.

### Human TRPM3 gene

**Fig. 1:**
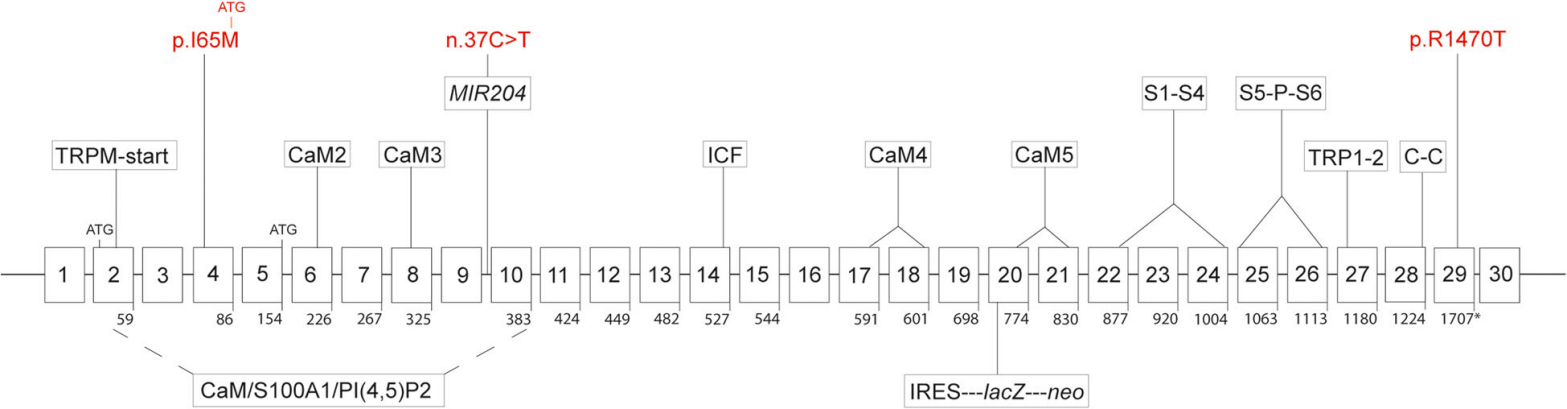
TRPM3_miR-204 gene organization and protein coding domains. Schematic of the human TRPM3 gene (*TRPM3*) coding for RefSeq transcript-variant 9 and channel-isoform k. The non-coding miR-204 gene (*MIR204*) is located in intron-9 of *TRPM3*. Exons are indicated by numbered boxes (1–30). Codon numbers are shown below each coding exon (exons 3, 9, 16, and 30 are skipped). ATG denotes alternative translation start-sites. Asterisk denotes translation stop-site. Mutations in *TRPM3* (exon 4 and 29) and *MIR204* (intron 9) underlying human eye disease are shown in red. The approximate locations of TRPM3 protein coding domains are indicated as follows. TRPM-start, consensus start of the N-terminal TRPM homology domain (~ 700 amino acids). CaM/S100A1/PI(4,5)P2, calmodulin, S100A1 Ca^2+^-binding protein, and phosphatidylinositol-4,5-biphosphate binding domains. CaM2–5, calmodulin binding domains 2–5. ICF, indispensable for channel function. S1-S4, transmembrane segments 1–4, S5-P-S6, canonical pore flanked by transmembrane segments 5 and 6. TRP1–2, TRP box/motif 1 and 2. C-C, coiled-coil domain. The insertion site of a gene-targeting construct (IRES-*lacZ*-*neo*) used to generate a null allele in the mouse TRPM3 gene (*Trpm3*) is located in exon 20

According to public genomic databases (e.g., National Center for Biotechnology Information, NCBI, Genome Reference Consortium Human Build 38 patch release 13, GRCh38.p13), the human TRPM3 gene (*TRPM3*) is one of the largest genes located on the long-arm of human chromosome 9 (9q) spanning over 0.9 Mb (Table 1). The NCBI *TRPM3* reference sequence (RefSeq) comprises at least 30 exons that undergo extensive alternative splicing to generate at least 23 alternative transcript variants (1–23) encoding 23 predicted protein isoforms (a-w) (Supplementary Table 1). The 23 RefSeq variants differ mainly in usage of exons 1, 2, and 5 as translation start-sites and usage of exons 4, 9, 16, and 18 that encode regions of the amino (N) terminal TRPM domain (Fig. 1). Of the 23 RefSeq transcripts, the longest variant (16) encodes a predicted 1744 amino acid isoform (o) and the shortest variants (7, 8, 13, 22) encode isoforms of 230 (h) to 385 (l) amino acids with the remainder ranging from 1184 (v) to 1719 (m) residues (Supplementary Table 1). Notably, the short variants (7, 8, 13, 22) encode N-terminal short ‘cytoplasmic’ isoforms (h, c, l, u) that terminate in exon-10 and do not encode the transmembrane channel region and other carboxyl (C) terminal domains raising the possibility of alternative non-channel functions.

**Table 1:** Genomic summary of the TRPM3 and miR-204 genes in humans (*TRPM3*, *MIR204*) and mice (*Trpm3*, *Mir204*)

Gene symbol	Gene ID	Cytogenetic locus	Physical locus	Gene size	Exon count	Transcript variants	Protein isoforms	Gene variants	Disease variant/disease model	Ocular phenotype (other)	Gene/phenotype MIM No.	Reference
Human (GRCh38.p13^a^)												
*TRPM3*	80036	9q21.12-q21.13	Chr9: 70529060–71,446,971, complement	917,911 kb	30	1–23 (RefSeq), X1-X8, X10-X13, X15, X18-X19 (predicted)	a-w (RefSeq), X1–8, X10–13, X15, X18-X19 (predicted)	~ 244,676 Total (220,391 SNVs, 4221 deletions, 1882 insertions, 974 CNVs)	p.Ile65Met	Cataract ± glaucoma and anterior segment defects	608961	81
*MIR204*	406987	9q21.12	Chr9: 70,809,975–70,810,084, complement	110 bp	1 (located in intron-9 of *TRPM3*)	miR-204-5p, miR-204-3p		~ 180 Total (~ 48 SNVs, ~ 97 CNVs)	n.37 C>T	Retinal dystrophy and iris coloboma ± congenital cataract (RDICC)	610942/616722	83
Mouse (GRCm38.p6 C57BL/6 J^b^)												
*Trpm3*	226025	19; 19 B	Chr19: 22137797–22,995,410	857,613 kb	33	1–30 (RefSeq) X1-X15, X18-X21 (predicted)	a-z, aa-dd (RefSeq), X1-X15, X18-X21 (predicted)	13,684 SNVs	*Trpm3*-null mouse	Attenuated pupillary light response (Noxious heat insensitivity)		46, 93
*Mir204*	387200	19; 19 B	Chr19: 22,750,605 - 22,750,672	68 bp	1 (located in intron-9 of *Trpm3*)	miR-204-5p, miR-204-3p		37 SNVs	*Pax6*-null mouse	Lens development		165

^a^GRCh38.p13—Genome Reference Consortium Human Build 38 patch release 13

^b^GRCm38.p6—Genome Reference Consortium Mouse Build 38 patch release 6

In addition to the 23 validated RefSeq transcripts, there are predicted to be at least 15 other *TRPM3* transcript variants (Supplementary Table 1). These differ mostly from the RefSeq transcripts either by inclusion of a non-coding exon (11a) or by starting translation in exon-12 (X1-X7). The predicted isoforms range from 410 (X8) to 1734 (X10) amino acid residues and all but the shortest one (X8) harbor a transmembrane channel domain. Once validated, *TRPM3* may encode at least 38 transcript variants and channel isoforms. In contrast to NCBI, the Ensemble genome browser currently supports 16 human TRPM3 transcripts/isoforms with high confidence (www.ensemble.org).

According to the NCBI Variation Viewer (GRCh38.p12), *TRPM3* harbors over 220,391 single-nucleotide polymorphisms/variants (SNPs/SNVs) and over 2254 structural variants including deletions (~ 4221), insertions (~ 1882), and copy number variants (CNVs, ~ 974). The vast majority of SNVs (> 92.3%) reside in the introns and flanking untranslated regions (5′-UTR, 3′-UTR). Over 1600 coding variants (cSNVs) have been reported in *TRPM3*. Most are non-synonymous including missense (~ 1048), nonsense (~ 43), splice-site (~ 43), and frameshift (~ 29) cSNVs of unknown pathogenic significance and the remainder are synonymous (~ 543) cSNVs (Table 1). While several non-synonymous cSNVs are predicted in silico to be damaging/pathogenic, so far only two missense mutations (p.I65M, p.R1307T) in *TRPM3* have been linked with inherited forms of human cataract [81, 82]. In addition, a SNV (rs9792446 G/A) located in intron-2 of *TRPM3* has been tentatively associated (*p* = 1.0e-7) with age-related nuclear cataract in a cohort of 2265 twins (age ≥ 50).

Beyond eye disease however, *TRPM3* variants have also been associated with diverse phenotypic traits or diseases in humans. Rare deletions involving coding exons of *TRPM3* have been documented in one case of Kabuki syndrome and in two brothers with autism [84, 85]. Common non-coding SNVs in *TRPM3* have been tentatively associated with longevity, elevated low-density lipoproteins and triglycerides, systemic sclerosis, aspirin-exacerbated respiratory disease, and thyroid nodules [86–90]. Coding variants in *TRPM3* (p.V83M, p.P937Q) have been speculated to cause intellectual disability and epilepsy; however, supporting functional expression studies were not performed [91]. Finally, a TRPM3 gene variant (*T/C) has been significantly associated with increased racing speed in the whippet breed of dogs accounting for 11.6% of the total variance in racing performance [92].

### Mouse TRPM3 gene

The mouse TRPM3 gene (*Trpm3*) is located on murine chromosome 19 and spans over 0.85 Mb comprising some 32–33 exons that generate at least 30 RefSeq transcript variants (1–30) encoding 30 protein isoforms (a-w, p, y, z, aa-dd) (Supplementary Table 2). Similar to *TRPM3*, alternative splicing mostly affects usage of exons 1, 2, and 5 encoding the N-terminus; however, there is currently no simple correlation between the human and mouse RefSeq transcript variants and channel isoforms. Of the 30 mouse RefSeq transcripts, the longest variant (10) encodes a predicted 1744 amino acid isoform (j) and the shortest variants (8, 9, 28–30) encode isoforms of 234–265 amino-acids (h, i, bb-dd) with the remainder ranging from 785 to 1734 residues (a-g, j-z, aa) (Supplementary Table 2). Like *TRPM3*, the shortest mouse RefSeq variants (8, 9, 28–30) encode N-terminal short isoforms (h, i, bb-dd) that prematurely terminate in exon-10 or exon-11 and do not encode the transmembrane channel region and other C-terminal domains. Surprisingly however, two other short mouse RefSeq variants are predicted to start translation in exon-22 (two isoforms p encoded by variants 16 and 24) or exon-26 (isoform aa) and encode all or part of the transmembrane-channel region and other C-terminal domains without the N-terminal domains.

In addition to the 30 mouse RefSeq transcripts/isoforms, 19 other *Trpm3* transcript variants have been predicted bringing the potential total of mouse TRPM3 isoforms to 49 (Supplementary Table 2). By contrast, the Ensemble genome browser currently supports 24 mouse TRPM3 transcripts/isoforms with high confidence (www.ensemble.org).

According to the Mouse Genome Informatics (MGI) database (build 142), *Trpm3* harbors over 13,646 validated SNVs across some 56 strains of inbred mice (http://www.informatics.jax.org/snp). The vast majority of *Trpm3* SNVs reside within introns (13,582, 99.5%) or mRNA UTRs (61). Of 18 cSNVs, 17 are synonymous across 49 inbred strains and only one (rs38720863 G/A) is non-synonymous in three of 14 inbred strains. This non-synonymous cSNV is predicted in silico to result in a conservative missense substitution (p.R1223H) with potentially damaging effects at the protein level (PolyPhen-2 score = 0.997, SIFT score = 0.00). While no spontaneous *Trpm3* mutant alleles or phenotypes have been documented, at least one targeted null allele of *Trpm3* has been reported [46] (Fig. 1). *Trpm3*-null mice are viable and fertile; however, they display an impaired response to noxious heat [46, 49, 50] and an attenuated pupillary light response [93].

### MiR-204 gene

**Fig. 2:**
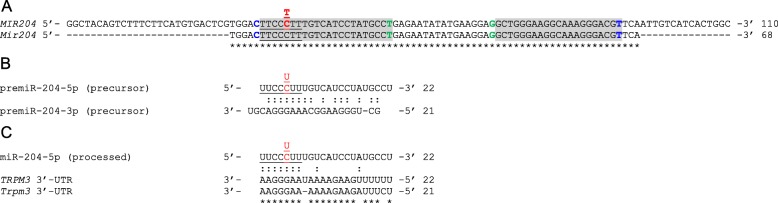
MiR-204 sequence, processing, and target sites. **a** Sequence alignment of human (*MIR204*) and mouse (*Mir204*) miR-204 genes. The 5p and 3p arms are shaded gray. The 7-nucleotide seed regions are underlined. Nucleotides critical for Drosha cleavage (C32, T92) and Dicer cleavage (T54, G71) are shown in blue and green, respectively. The n.37C > T transition underlying RDICC is shown in red. **b** Precursor (pre)miR-204 stem formation between the 5p and 3p arms. **c** Processed miR-204-5p aligned with binding sites in the mRNA 3′-untranslated regions (UTRs) of human *TRPM3* and mouse *Trpm3*. Asterisks (*) denote identical nucleotides. Colons (:) denote base pairing. Dashes (−) denote sequence gaps

Both *TRPM3* and *Trpm3* harbor non-coding microRNA genes that are co-transcribed in the same direction as the host gene and participate in the post-transcriptional regulation of gene expression. *MIR204* (110 bp) is located in intron-9 of *TRPM3* on human chromosome 9 and *Mir204* (68 bp) is located in intron-9 of *Trpm3* on mouse chromosome 19 (Table 1). *MIR204* and *Mir204* are highly conserved with identical seed regions required for binding of the processed miR-204 (5p) product to mRNA transcripts of target genes that include *TRPM3* and *Trpm3*, respectively [56] (Fig. 2). Notably, miR-204 and miR-211, which is located within an intron of the TRPM1 gene, share the same seed region and have been classified as one microRNA family with the same set of predicted target genes [94] (www.targetscan.org).

The NCBI Variation Viewer lists 180 variants in *MIR204* including CNVs (~ 97) and SNVs (~ 48), whereas the MGI database lists 37 SNVs in *Mir204* across 19 inbred mouse strains. One *MIR204* SNV (rs767146880) that lies within the seed region (n.37C>T) has been linked with inherited retinal dystrophy and iris coloboma [83]. Recently, another SNV (rs718447 A/G) flanking *MIR204* upstream coupled with low expression of miR-204 has been identified as a risk factor for poor clinical outcome in acute myeloid leukemia [95] supporting a role for miR-204 in tumor suppression [96].

## TRPM3 channel structure-function domains

**Fig. 3:**
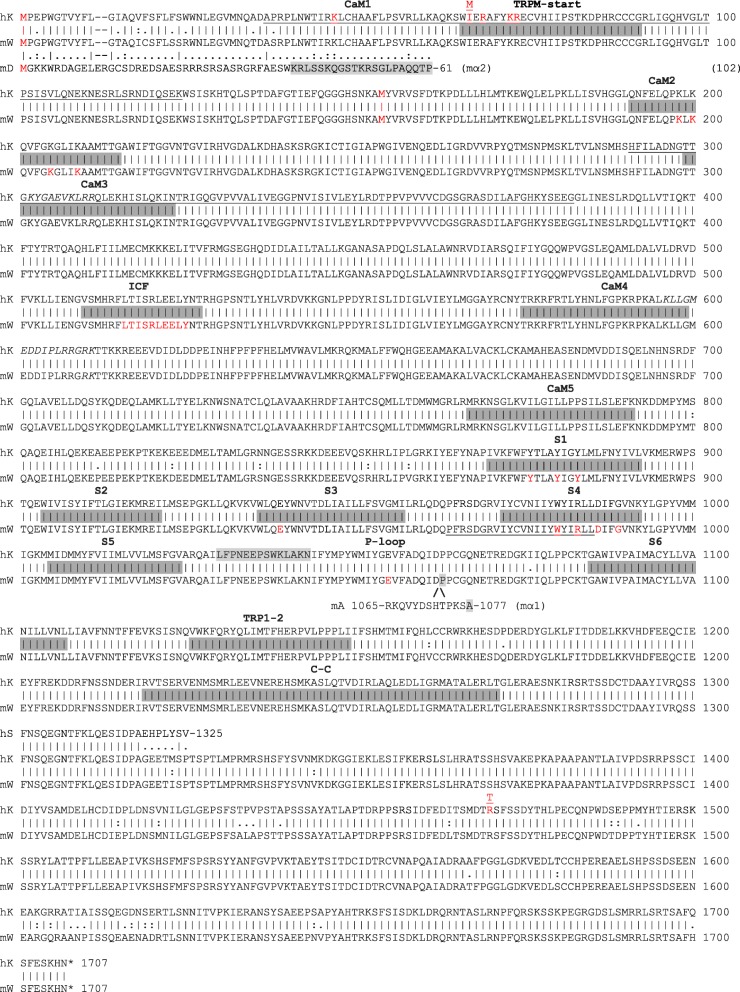
Amino acid alignment of human TRPM3 isoform-k (hK) and mouse TRPM3 isoform-w (mW). Partial amino acid alignments of mouse TRPM3 isoform-d (mD, alias mα2) and isoform-a (mA, alias mα1) are included. Bars denote identical amino acids. Colons denote similar amino acid changes. Single dots denote dissimilar amino acid changes. Dashes denote gaps. CaM1, first calmodulin binding-site in mD/mα2 (K41-P61, shaded gray). Gray-shaded bars denote protein domains as follows. TRPM-start, consensus start-motif of the ~ 700 amino acid TRPM domain. CaM2–5, calmodulin binding-sites 2–5 in hK, mW, and mD/mα2. An in silico CaM6 binding-site (P966-L987) overlapping S4 in mW is underlined. CaM/S100A1 and PI(4,5)P2 binding-sites in hK (A35-K124, H291-G382) are also underlined. Putative PH-like domains and PI(4,5)P2 binding-sites (K302-R311, K596-K611) in hK are shown in italics. ICF, indispensable for channel function domain (mD/α2), S1-S6, transmembrane α-helical segments, P-loop, canonical pore-forming loop, TRP1–2, TRP-domain containing TRP-box 1 (1127-WKFQR-1132) and TRP-box 2 (1144-LPPPL-1148), and C-C, coiled-coil domain (R1219-T1271). **/\** indicates location of ‘long-pore’ region of mA/α1 that is absent in hK, mW, and mD/mα2. Alternative methionine translation start-sites (M1, M65, M154), cataract-associated mutations (p.I65M, p.R1470T), and functionally important amino-acids in the CaM/S100A1 binding-site (K45, R67, K71, R72), CaM2 binding-site (K198, K200, K205, K209), ICF domain (L516-Y525), S1 helix (Y976, Y780, Y783), S3 helix (E939), S4 helix (W980, R983, D986, G989), and P-loop region (E1055) are shown in red font. A synthetic-peptide (L1030-N1043) located in the third extracellular loop (P-loop) of hK used to raise a polyclonal antibody (TM3E3) is shaded gray. The C-terminal sequence of the truncated hTRPM3–1325 isoform (hS) is indicated. Asterisks denote translation stop-sites

Like other TRP channels, TRPM3 channels share a predicted multi-pass (type-2) transmembrane topology, assembled from tetramers of identical (homomeric) or similar (heteromeric) TRP-subunits around a central pore, characteristic of the voltage-gated ion channel superfamily [58, 97, 98]. In addition to forming homo-tetramers, TRPM3 and TRPM1 subunits are believed to form heteromeric channels [63]. Each TRPM3 subunit possess cytoplasmic amino- (N-) and carboxy- (C-) terminal domains that flank the transmembrane (TM) domain containing six cell membrane-spanning α-helices/α-helical segments (S1-S6) joined by three extracellular and two intracellular loop regions. The neurosteroid agonist pregnenolone sulfate (PS) is believed to bind to a conserved stereo-specific (chiral) protein binding site(s) (referred to as the ‘steroid modulatory domain’) that likely resides within the four extracellular-transmembrane regions of TRPM3 channels [40, 60, 61]. The TM domain may be further sub-divided into two functional cation-conducting pore domains. First, the central, canonical cation-conducting pore of TRPM3-tetramers is formed by α-helices S5 and S6 interconnected by the hydrophobic pore-forming loop (P-loop) (Fig. 3). A polyclonal antibody (TM3E3) directed against a conserved 14-amino acid synthetic-peptide (L1030-N1043) located in the third extracellular loop (E3) of TRPM3 has been reported to elicit specific but incomplete inhibition of recombinant hTRPM3 channel activation independent of the agonist used [99] (Fig. 3). Second, the non-canonical cation-conducting pore believed to be contained within the S1-S4 ‘bundle’ analogous to the voltage-sensor domain (VSD) of voltage-gated K^+^, Ca^2+^, and Na^+^ ion channels (Fig. 3). Like other TRPM channels, TRPM3 lacks the N-terminal ankyrin repeats, found in the TRPA, TRPC, and TRPV sub-families, and instead display a unique, long N-terminal region of ~ 700 amino-acids referred to as the TRPM homology domain that starts with the consensus motif (W/F)IX_3_(F/L/I)CK(R/K)EC(V/I/S)X_12-24_CXCG [98] (Fig. 3).

Multiple binding sites for the Ca^2+^-binding protein calmodulin (CaM), which harbors 4-EF hand structures, have been identified within the N-terminal TRPM homology region of TRPM3 [98, 100, 101]. Two N-terminal regions of ~ 90 amino-acids, between A35-K124 and H291-G382, in human TRPM3 have been experimentally shown to bind both CaM and dimers of the structurally similar S100 protein, S100A1, [100] (Fig. 3). In vitro mutagenesis revealed that positively charged amino acids (K and R residues) within these regions were critical for CaM/S100A1 binding (Fig. 3). These CaM/S100A1 binding sites also serve as high-affinity binding sites for the cell-membrane phosphatidylinositol phosphate (PIP), phosphatidylinositol 4,5-biphosphate (PI(4,5)P2) [102]. Up to five shorter CaM-binding sites (25 amino-acids/residues) between K41-S65 (CaM1), Q193-G217 (CaM2), T301-N325 (CaM3), T577-G601 (Cam4), and M769-K793 (CaM5) have been experimentally validated in mouse TRPM3 (α2 isoform) [101]. Two of these mouse TRPM3α2 CaM-binding sites (CaM1/K41-S65, CaM3/T301-N325) overlap with the human TRPM3 CaM/S100A1-binding sites (Fig. 3). The mouse CaM2 and CaM3 binding sites are encoded by single coding exons present in all TRPM3 isoforms, whereas, CaM1, CaM4 and CaM5 binding sites overlap alternatively spliced exons (Fig. 3**,** Supplementary Tables 1 and 2). Further, the CaM3 and CaM4 binding sites overlap pleckstrin homology (PH)-like domains containing a consensus motif of positively charged (basic) amino-acids—(K/R)-X_n_-(K/R)-X-(K/R)_2_—that also serve as putative PI(4,5)P2 binding sites [103] (Fig. 3). Pull-down assays revealed that CaM binding to TRPM3α2 was Ca^2+^-dependent and in vitro mutagenesis studies determined that four positively charged lysine (K) residues within CaM2 binding-site (Q193-G217) are critical for both CaM binding and overall TRPM3 channel stability [101]. A putative sixth CaM-binding site in TRPM3-α2 has been identified in silico, (P966-L987); however, this site overlaps the S4 transmembrane domain and is likely masked to CaM-binding during canonical pore opening [101] (Fig. 3).

Toward the C-terminus, TRPM (and TRPC) sub-family members contain a unique 23–25 amino acid motif referred to as the TRP-domain, containing TRP-box 1 (WKFQRY) and a proline-rich region referred to as TRP-box 2 [18, 29]. The TRP-domain of TRPM3 also contains a series of basic K/R residues (KFQRYQLIMTFHER) similar to that required for PIP binding by TRPM5 and TRPM8 [103, 104]. Further C-terminal to the TRP-domain lies a conserved coiled-coil (CC) domain (R1219-T1271) (Fig. 3) harboring a heptad repeat pattern of hydrophobic and polar or charged amino acid residues that is believed to be sufficient for tetramer self-assembly and necessary for channel function [29, 58, 105, 106]. However, TRPM3 (and TRPM1) lacks the C-terminal enzyme domains found in the TRPM2 (pyrophosphatase), TRPM6 (kinase), and TRPM7 (kinase) channels or so-called chanzymes [58, 107].

### Canonical TRPM3 cation pore (S5-P-S6)

Functional expression studies in transfected cells (mostly HEK293) or in cultured primary cells (e.g., β cells, neurons), coupled with electrophysiological (e.g., patch-clamp) recording and fluorometric Ca^2+^ imaging techniques have been used to characterize the permeability and gating properties of the central canonical TRPM3-cation pore ('alpha-pore') in the absence or presence of known agonists including PS and/or heat [47, 48, 50, 51, 57–62]. Two human kidney-derived TRPM3 isoforms (hTRPM3_1555 and hTRPM3_1325 amino-acids) were the first to be characterized as spontaneously or constitutively active Ca^2+^ influx channels located in the cell membrane and were inhibited, non-specifically, by trivalent lanthanides La^3+^ and Gd^3+^ [108, 109]. Spontaneous Ca^2+^ influx by hTRPM3a_1555 was stimulated by passively depleting Ca^2+^ stores with thapsigargan or by activation of muscarinic receptors using carbachol [108]. Constitutive Ca^2+^ influx by hTRPM3_1325 that was activated by extracellular hypotonic stress (200 mOsmol) caused cell swelling consistent with a role in cell volume-regulation [109]. Similar overexpression studies subsequently confirmed that two alternatively spliced mouse brain TRPM3 isoforms (mTRPM3α1 and mTRPM3α2) functioned as spontaneous, weakly voltage-dependent, Ca^2+^ influx channels with outwardly rectifying current-voltage (I-V) relationships [110]. Significantly, these studies were the first to reveal that alternative splicing of a 12-amino acid motif (R1065-S1076) located within the canonical pore (P-loop) region (Fig. 3) dramatically influenced permeability of mTRPM3α1 and mTRPM3α2 to divalent cations (Ca^2+^ and Mg^2+^) [110]. The ‘short-pore’ α2 isoform (lacking R1065-S1076) was > 10-fold more permeable to Ca^2+^ and > 100-fold more permeable to Mg^2+^ than the ‘long-pore’ α1 isoform (containing R1065-S1076). Additionally, extracellular Na^+^ inhibited mTRPM3α2 channels but not mTRPM3α1 channels, whereas intracellular Mg^2+^ blocked both canonical channels. In addition to Ca^2+^ and Mg^2+^, mTRPM3α2 channels were found to provide a regulated influx pathway for Zn^2+^ ions in vitro [59]. By contrast, homomeric TRPM1 channels and heteromeric TRPM1/TRPM3 channels were inhibited by extracellular Zn^2+^ in vitro [63, 111].

Subsequently, comparative expression of another mouse brain splice-variant (mTRPM3α7) lacking an 18-amino acid region (V512-T529) that lies N-terminal to the canonical pore of mTRPM3α2, has led to the identification of a key functional domain in TRPM3 [112]. Homomeric α7-channels were non-functional and heteromeric α2/α7-channels exhibited significantly reduced (> 70%) channel number at the cell membrane and decreased agonist-induced Ca^2+^ influx compared to homomeric α2-channels. Site-directed mutagenesis of this mTRPM3α2 sequence has further defined a conserved, 10-amino acid, leucine-rich domain (L518-Y527) that was indispensable for channel function (ICF domain, Fig. 3) [112]. Consequently, the ICF domain is believed to facilitate correct tertiary folding of TRPM subunits and formation of functional homo-/hetero-tetrameric TRPM channels [112]. Together, these studies suggest that alternative splicing inside and outside the canonical TRP-pore region can directly influence TRPM3 channel permeability and that co-expression of channel-negative or short ‘cytoplasmic’ isoforms may serve as ‘decoy-receptors’ to modulate or fine-tune full-length TRPM3 channels [58, 98, 110, 112]. By analogy, short, cytoplasmic, N-terminal isoforms of TRPM1 (TRPM1-S) have been proposed to interact with full-length TRPM1 isoforms (TRPM1-L) in order to suppress channel activity of the latter by inhibiting its translocation to the plasma membrane in vitro [113].

### Non-canonical TRPM3 cation pore (S1–S4)

Recently, a second, distinct cation conductance pore has been discovered in native mTRPM3α2 channels that is analogous to the ‘gating-pore’ or ‘omega-pore’ associated with artificial and disease-causing mutations in the voltage-sensor domain (S4-helix) of classical voltage-gated K^+^, Ca^2+^, and Na^+^ channels [42, 114–116]. Simultaneous opening of the canonical and non-canonical pores was achieved by co-stimulation with PS and the antifungal agent clotrimazole (Clt) or its structural analogs (e.g., tamoxifen) or by administration of a synthetic Clt-like ligand CIM0216 that represents the most potent and specific agonist known for TRPM3 channels [42, 114]. In contrast to the canonical pore (S5-P-S6), activation of the non-canonical pore (S1–S4) resulted in distinct conductance and gating properties including (1) induction of currents with an I-V relationship that rectifies in both inward and outward directions, with marked inward currents at strong hyperpolarizing membrane potentials; (2) a single-channel conductance of ~ 20 pS (versus ~ 50 pS for canonical pore); (3) low Ca^2+^ high Na^+^ permeability; and (4) resistance to Ca^2+^-dependent de-sensitization and inhibition by La^3+^. Further support for a non-canonical TRPM3 pore has been provided by site-directed mutagenesis of functionally critical amino acids in TRPM3 identified largely by homology modeling with voltage-gated K^+^-channels. First, in vitro mutagenesis of a positively charged glutamate within the P-loop region (E1057C) inhibited activation of the canonical pore but not the non-canonical pore. Second, serial replacement of four amino acids (W982, R985, D988, or G991) located within the S4-helix of mTRPM3α2 (Fig. 3) inhibited activation of the non-canonical pore but not the canonical pore [116]. Notably, these four TRPM3 residues aligned with the four positively charged arginine residues (R1–R4) in the S4-helix of K^+^-channels that are critical for voltage sensing. Third, serial neutralization of three aromatic, tyrosine residues (Y878, Y882, Y885) located in the S1-helix of mTRPM3α2 (Fig. 3), which are believed to have proximity with W982 in the S4-helix, resulted in non-canonical pore inhibition. Fourth, serial neutralization of negatively charged residues (E941Q, D964N) located in the S3-helix also inhibited activation of the non-canonical pore. Altogether, these data point to key roles for the S1, S3, and S4 α-helices in TRPM3 non-canonical pore formation. In particular, substitution of larger, charged R residues with smaller, uncharged amino acids (W, D and G) at the R1, R3, and R4 positions of the S4-helix in K^+^-channels appears to facilitate non-canonical pore opening in TRPM3 channels.

## TRPM3 channel signal transduction

**Fig. 4:**
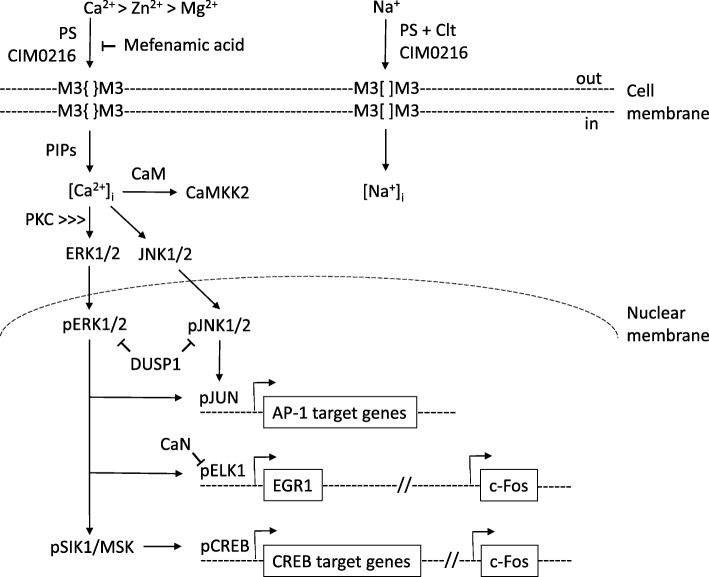
Schematic summary of TRPM3 channel gating and signal transduction. Agonists PS or CIM0216 stimulate Ca^2+^ influx via the canonical central pore (S5-P-S6) of TRPM3 homomeric tetramers ({}), whereas, mefenamic acid acts as an antagonist. Membrane phosphoinositides (PIPs) enhance PS-activated TRPM3 Ca^2+^ influx. PS in combination with Clt, or CIM0216 by itself, can also stimulate Na^+^ influx via an alternative pore opening ([]), distinct from the canonical TRP-pore. TRPM3 and TRPM1 monomers can also form heteromeric channels. TRPM3 channels are also permeable to Zn^2+^ and Mg^2+^, whereas, Zn^2+^ inhibits TRPM1 channels. TRPM3-elevated intracellular Ca^2+^ phospho-activates cytoplasmic MAPK signal transducers ERK1/2 and JNK1/2 that in turn phospho-activate nuclear transcription factors AP-1, ELK1, and CREB. Phosphatases CaN and DUSP1 provide feed-back inhibition of TRPM3-dependent Ca^2+^ signaling by de-phosphorylation of transcription factor pELK1 and the MAPKs pERK2 and pJNK1/2, respectively

Activation of TRPM3 channels at the cell membrane (HEK293 cells) has been shown to trigger a Ca^2+^-dependent intracellular signaling cascade of mitogen-activated protein kinases (MAPKs) and several nuclear phosphatases that regulate the stimulus response transcription factors, including the activator protein-1 (AP-1) transcription factor complex, resulting in altered gene expression patterns (Fig. 4).

### Kinases

PS-stimulation of recombinant hTRPM3 channel (1325 amino acids) Ca^2+^ influx results in activation (i.e., phosphorylation) of two key MAPKs that mediate signal transduction from the cytoplasm to the nucleus/nuclear transcription factors [117]. First, hTRPM3 Ca^2+^ influx activates extracellular signal-regulated protein kinase 1 (ERK1/MAPK2) and 2 (ERK2/MAPK1) most likely via upstream action of the protein kinase C (PKC) family of serine/threonine protein kinases and the c-Raf proto-oncogene serine/threonine-protein kinase (RAF1) or MAP kinase-kinase-kinase (MAP3K) [117]. Second, an hTRPM3-mediated rise in intracellular Ca^2+^ results in phosphorylation of c-Jun N-terminal protein kinase 1 and 2 (JNK1/2) or MAPK8 and MAPK9, respectively. In addition, hTRPM3 channel Ca^2+^ influx activated Ca^2+^/calmodulin-dependent protein kinase-kinase-2 (CAMKK2) that in turn phospho-activated Ca^2+^/calmodulin-dependent protein kinases (CAMKs) [56, 117].

### Transcription factors

At least three transcription factors have been identified as nuclear targets for phosphorylated forms of JNK1/2 (pJNK1/2) and ERK1/2 (pERK1/2) resulting from PS-activation of hTRPM3 Ca^2+^ influx [117–120]. A key target for pJNK1/2 is the basic-region leucine-zipper (bZip) transcription factor c-Jun proto-oncogene (JUN)—a major component of the AP-1 transcription factor complex. AP-1 is a collection of homo-dimers and hetero-dimers of the JUN, c-Fos proto-oncogene (FOS), c-Maf proto-oncogene (MAF), and activating transcription factor (ATF) sub-families of bZip transcription factors that serves as an intersection point for multiple intracellular signaling cascades involved in the regulation of cell proliferation, transformation, differentiation, and death in a tissue-specific manner [121]. For pERK1/2, a key nuclear target is the erythroblast transformation specific (ETS) domain-containing protein Elk-1 (ELK1) [122–124]. ELK1 belongs to the ternary complex factor (TCF) sub-family of ETS transcription factors that in complex with a dimer of the serum response factor (SRF) interacts with the serum response element (SRE) to regulate transcription of downstream target genes including the zinc-finger transcription factor early growth response protein-1 (EGR1) and FOS. In addition, pERK1/2 phosphorylates the serine/threonine protein kinase salt-inducible kinase-1 (SIK1) that in turn phosphorylates the bZip transcription factor cAMP response element binding protein (CREB) thereby activating downstream target genes of CREB including FOS and possibly that for calcitonin; calcitonin gene-related peptide 1 (CGRP1). Ultimately, phospho-activation of the stimulus response transcription factors, AP-1 (JUN, FOS), CREB, ELK1, and EGR1, leads to upregulation of mostly uncharacterized delayed response genes including that for the inflammatory response chemokine interleukin-8 (IL8) [125].

### Phosphatases

At least two nuclear phosphatases have been implicated in downregulating transcriptional activation resulting from PS-stimulated hTRPM3 Ca^2+^ influx [117, 123]. First, hTRPM3 Ca^2+^ influx activates the MAP kinase or dual specificity (tyrosine/threonine) phosphatase-1 (MKP1/DUSP1) that negatively regulates the TRPM3 signaling cascade by de-phosphorylating pERK2/MAPK1 and pJNK1/2. Second, the Ca^2+^/calmodulin-dependent serine/threonine protein phosphatase calcineurin (CaN) inhibits the TRPM3 signaling cascade by de-phosphorylating pELK1 [117, 123]. CaN is composed of two polypeptides the protein phosphatase 3 catalytic subunit-alpha (PPP3CA) and the protein phosphatase 3 regulatory subunit-beta (PPP3R1) and mutant forms of both CaN subunits have been shown to inhibit AP-1 regulated transcription activated by hTRPM3_1325_ Ca^2+^ influx. Overall, the concerted action of DUSP1 and CaN phosphatases acts as a negative feed-back loop for TRPM3-specific Ca^2+^-dependent signal transduction.

In addition to PS, CIM0216-activation of hTRPM3_1325_-channel Ca^2+^ influx was also reported to activate AP-1 and upregulate the transcriptional activation potential of JUN and FOS [126]. Although CIM0216 was more potent than PS at simulating hTRPM3_1325_-channel Ca^2+^ influx, it was less effective (~ 50%) than PS at AP-1 activation and similarly effective as PS in stimulating c-JUN and c-FOS, whereas mefenamic acid from the fenamate class of NSAIDs was completely inhibitory [118, 126]. Such transcriptional activation and inhibition evoked by recombinant hTRPM3_1325_-channels in vitro appears to be physiologically relevant since PS-stimulation of endogenous TRPM3 channels in rat insulinoma (INS-1) cells or primary mouse islet cells also led to ERK1/2 activation and enhanced expression of EGR1 and the AP-1 complex [127, 128].

## Ocular expression and regulation of TRPM3_miR-204

An expressed sequence tag (EST) for TRPM3 (alias MLSN2) was first isolated from a post-mortem human iris cDNA library and similar TRPM3 ESTs were found in human lens, retina, and retinal pigment epithelium/choroid cDNA libraries present in the NEIBank sequence database [129, 130] (https://neibank.nei.nih.gov). Subsequent ocular expression of TRPM3 and/or its hosted miR-204 have been detected, mostly by transcript PCR amplification, in situ hybridization, or microarray based techniques, in several human and mouse ocular tissues, notably ocular epithelial cells (Supplementary Table 3) (http://biogps.org). Currently, unbiased, high-throughput RNA-sequencing techniques have begun to provide more quantitative ocular expression profiles for TRPM3 and miR-204 in human and mouse eye tissues [131–134] (Supplementary Table 3).

### Human TRPM3_MIR204

In the human eye, multiple RefSeq *TRPM3* transcript variants were present in the lens [81] and TRPM3 transcript levels in a human adult RPE cell line (ARPE-19) approached those found in native RPE cells [135]. Recently, TRPM3 has been immuno-localized to sub-domains of the apical plasma membrane of human fetal RPE cells with particular enrichment at apical tight junctions and the base of primary cilia [136]. RNA sequencing confirms that TRPM3 is more abundant in human RPE tissue and cell lines and a lens stem cell line than in retinal or corneal derived cells [137, 138].

*MIR204* transcripts have been detected in the human ciliary body, trabecular meshwork (HTM) cells, and in lens epithelium and established human lens epithelial cell lines (HLE-B3, SRA01/04) [83, 139–143]. MicroRNA sequencing ranked miR-204 as the most abundant miRNA in human ciliary body and the fourth most abundant of the 11 miRNAs accounting for ~ 80% of normalized reads collectively expressed in human ciliary body, cornea, and trabecular meshwork—ocular tissues involved in glaucoma and keratoconus [134]. In human retina, miR-204 is expressed in the ganglion cell layer (GCL), inner nuclear layer (INL), and outer nuclear layer (ONL) along with the retinal pigment epithelium (RPE) and the ARPE-19 cell line [83, 139, 144, 145]. MicroRNA sequencing analysis ranked miR-204 as the fifth most highly expressed miRNA in human retina and the most abundant miRNA in human RPE/choroid [133].

### Mouse Trpm3_Mir204

In mouse anterior eye tissues, *Trpm3* transcripts are expressed in ciliary body and lens epithelium [93, 146, 147] and in mouse lens epithelial cell lines [148, 149]. RNA sequencing established that *Trpm3* transcripts were enriched in the mouse lens during embryonic development (E10.5-E16.5) [150]. In mouse retina, *Trpm3* transcripts are expressed in the optic-cup during eye development and in mature retina *Trpm3* transcripts are present in sub-populations of cells in the GCL (Muller cell end-feet) and INL (Muller cell bodies), and in the RPE and optic nerve head (ONH) [93, 147, 151, 152]. TRPM3 was also strongly immuno-localized to the inner plexiform layer (IPL) and a subset (~ 40%) of cells in the GCL with weak immuno-labeling of the INL and outer plexiform layer (OPL) of mouse retina [153]. Quantitative PCR revealed that *Trpm3* transcripts represented the second most abundant *Trpm* transcript in mouse optic nerve head (ONH), whereas qPCR and immuno-localization ranked TRPM3 channels as the main TRP channel in optic nerve glial cells (astrocytes and oligodendrocytes) [152, 154].

*Mir204* transcripts have been localized to mouse ciliary body non-pigmented epithelium, iris, lens epithelium, and corneal epithelium [139, 155–158]. MicroRNA sequencing ranked miR-204 as the second most abundant miRNA (after miR-184) during lens development (E15-P9) with miR-204-5p representing the dominant form of processed miR-204 [132]. In mouse retina, *Mir204* transcripts are widely expressed in sub-populations of cells in the GCL, particularly Muller glia, INL, and in the RPE [93, 139, 146, 147, 151, 155–157, 159–163]. MicroRNA sequencing analysis ranks miR-204 as the sixth most highly expressed miRNA in the mouse retina and the fourth most abundant in the mouse RPE/choroid [131].

### Transcription factor regulation

Transcriptional regulation of TRPM3_miR-204 expression has been directly linked with at least two transcription factors important for vertebrate eye development. In mice, *Trpm3* and *Mir204* were found to be co-regulated by the paired-box 6 transcription factor, *Pax6*, during lens development and an evolutionarily conserved mechanism has been described in Japanese rice fish or medaka [164–167]. Disruption of *Pax6* resulted in downregulation (> 2-fold) of *Trpm3* and *Mir204* expression in the embryonic mutant lens, optic cup, and iris/ciliary body progenitors, consistent with direct *Pax6* activation of *Trpm3_Mir204* during eye development. Chromatin immunoprecipitation (ChIP), electrophoretic-mobility shift (EMSA), and reporter-gene transfection assays combined with in silico predictions have confirmed that *Pax6* directly binds to 5′-regulatory sequences upstream of *Trpm3* resulting in upregulation of both *Trpm3* and *Mir204* transcripts [165–167].

In addition to *Pax6*, the microphthalmia/melanogenesis-associated transcription factor (MITF), which harbors basic helix-loop-helix (bHLH) and basic-region leucine zipper (b-Zip) ‘dimerization’ domains, has been implicated in regulation of TRPM3_miR-204 and TRPM1_miR-211 [31, 32, 145]. In human fetal (hf) RPE cells, siRNA-mediated knockdown of MITF resulted in downregulation of *TRPM3_MIR204* (and *TRPM1_MIR211*) along with other RPE differentiation genes (e.g., *TYR* and *TYRP1*). Conversely, co-transfection of MITF siRNA and pre-miR-204/211 prevented hf-RPE de-differentiation consistent with a critical role for MITF-mediated upregulation of miR-204/-211 in directing hf-RPE differentiation [145].

## Ocular function and dysfunction of TRPM3_miR-204

The divergent roles of miR-204 and TRPM3 in ocular development and disease, particularly of the lens and retina, have begun to emerge from loss-of-function (knock-out/down) or gain-of-function (e.g., overexpression) approaches using several model organisms and cell-culture systems.

### TRPM3_miR-204 in lens development and cataract

#### MiR-204 in lens development

An early indication that miR-204 was involved in eye development emerged from studies of lens regeneration in the Japanese newt [168]. Following lens removal in adult newts, dorsal (but not ventral) iris pigment epithelial cells transdifferentiate into lens-forming cells. Microarray analysis of this process revealed that miR-204 levels were upregulated (~ 2.7-fold) in the dorsal versus the ventral iris consistent with differential regulation of miR-204 target gene expression during lens development [168].

In medaka (*Oryzias latipes*, ola), miR-204 has been found to be critical for lens development by modulating repression of the ‘three amino acid loop extension’ (TALE) homeobox transcription factor myeloid ecotropic-viral integration site-1 homolog 2 or *Meis2* [164]. Morpholino antisense oligo-mediated knockdown of miR-204, by injection of fertilized one-cell medaka embryos, resulted in a morphant eye phenotype with incomplete penetrance characterized by microphthalmia (≥ 60% penetrance) and lens dysgenesis (in 90% of small eyes) [164]. Overexpression of miR-204 double-stranded mimic (agomiR) in medaka embryos also resulted in microphthalmia and lens dysgenesis [164, 169]. At the transcript level, morpholino inactivation of miR-204 resulted in abnormal activation of the transcription factor *Meis2*, whereas overexpression of miR-204 resulted in decreased *Meis2* levels in medaka embryo eyes. However, co-injection of a morpholino against *Meis2* partially rescued the morphant miR-204 eye phenotype, whereas targeted disruption of *miR*-*204*-*Meis2* interaction resulted in a morphant miR-204-like eye phenotype. *Meis2* was co-expressed with miR-204 in the medaka embryo lens and peripheral optic cup and contains a phylogenetically conserved 3′-UTR binding-site for miR-204 consistent with a direct target gene for miR-204 repression. Furthermore, *Meis2* has been shown to directly activate *Pax6* expression during eye development in zebrafish, chick and mouse embryos [164]. Taken overall, these data indicate that miR-204 suppression of the *Meis2*-*Pax6* pathway participates in the control of lens morphogenesis.

Beyond *Meis2*, a second target gene for miR-204 repression that encodes a member of the ankyrin repeat domain-containing protein family *Ankrd13a* has been identified during medaka lens development [170]. Morpholino knockdown of miR-204 led to abnormal lens morphogenesis with aberrant dorso-ventral organization of lens epithelium and lens fiber cells and disorganized fiber cells at the center of the lens vesicle. In addition, miR-204 inactivation caused mesenchymal neural crest cell mislocalization, whereas miR204 overexpression resulted in promotion of neural crest cell and lens cell migration and elongation.

Knockdown of miR-204 in a human lens cell line (H36CE) caused cell rounding accompanied by dramatic cytoskeleton changes including actin disassembly, radial distribution of microtubules, and increased focal adhesion formation further supporting a role for miR-204 in cell migration/adhesion. In silico screening of predicted miR-204 target genes identified *Ankrd13a* as a strong candidate gene that is expressed in the lens and predicted to interact with actin-binding and focal adhesion proteins. At the transcript level, morpholino knockdown of miR-204 resulted in upregulation of *Ankrd13a*, whereas overexpression of miR-204 resulted in decreased *Ankrd13a* levels in medaka embryo eyes. *Ankrd13a* was co-expressed with miR-204 in the medaka embryo lens and migrating neural crest cells and *Ankrd13a* harbored an evolutionarily conserved 3′-UTR binding-site for miR-204 suggesting that it was a direct target gene for miR-204 repression. Furthermore, co-injection of morphilinos against miR-204 and *Ankrd13* largely rescued (> 80%) the abnormal lens fiber cell dysgenesis found in the miR-204 morphant embryo but failed to rescue the accompanying aberrant dorso-ventral polarity of the lens epithelium and fiber cells. These data suggest that *Ankrd13* is part of a miR-204 repression network regulating vertebrate lens morphogenesis [170].

In mice, miR-204 upregulation by *Pax6* provides an important cue for lens development [165]. In silico analysis of genes that were upregulated (> 1.5-fold) in mouse lenses conditionally null for *Pax6*, revealed over-representation of genes with conserved miR-204 binding sites located in their 3′-UTR regions suggestive of good candidates for miR-204 repression during lens development. These predicted miR-204 target genes included a member of the sex determining region Y (SRY)-related high-mobility group (HMG) box family of transcription factors—*Sox11*—that is required for neurogenesis and ocular development along with several other genes involved in cell migration/motility including those for unconventional myosin-X (*Myo10*) and fibrillin-2 (*Fbn2*). Transfection studies confirmed that miR-204 bound to the 3′-UTR of *Sox11* transcripts and downregulated their translation in mouse neuroblastoma (Neu-2a) and human lens (H36CE) cell lines. In situ hybridization revealed that *Sox11* transcripts were elevated in conditional *Pax6*-null mouse lenses consistent with *Pax6* acting as an indirect negative regulator of *Sox11* expression via miR-204 repression. Transfection studies also confirmed that *Myo10* and *Fbn2* were downregulated by overexpression of miR-204 mimic and upregulated by miR-204 inhibitor. In medaka embryos, morpholino knockdown of miR-204 resulted in upregulation (21–38%) of *Sox11*, *Fbn2*, and *Myo10* transcripts, whereas overexpression of miR-204 mimic drove downregulation (21–43%) of these transcripts. Similarly, injection of medaka embryos with *Pax6* transcripts significantly downregulated (43–56%) transcripts for *Sox11*, *Myo10*, and *Fbn2*, whereas co-injection of miR-204 morpholino resulted in partial recovery (18–28%) of these transcript levels. Combined, these data demonstrate that *Pax6* simultaneously represses multiple genes involved in neurogenesis (*Sox11*) and cell motility (*Myo10*, *Fbn2*) through direct upregulation of *Mir204* thereby promoting a non-neuronal lens epithelial cell fate [165]. By extension, since miR-204 represses *Meis2* expression in the medaka eye [164] and *Meis1*/*2* regulates *Pax6* during mouse lens induction [164, 171], a negative feedback loop between murine *Meis1*/*2*, *Pax6*, and *Mir204* has been proposed [165]. However, *Meis1*/*2* expression is lost in the mouse lens by embryonic-day (E) 12.5, whereas *Pax6* and *Mir204* expression is retained throughout eye development suggesting that such a negative feedback loop may be restricted to early lens placode and/or vesicle formation [165].

#### MiR-204 in cataract

In addition to lens development, differential regulation of miR-204 has been associated with loss of lens transparency or cataract formation in humans and mice, including age-related cataract, congenital cataract, diabetic cataract**,** and secondary (post-surgical) cataract or posterior capsular opacification (PCO)—a post-operative complication of cataract surgery [172–177]. Microarray analysis has revealed downregulation (> 2-fold) of miR-204-5p and miR-204-3p in central, anterior lens epithelium (capsulorrhexis) samples from patients undergoing age-related cataract surgery [173, 175] and miR-204-5p was one of the five most downregulated (~ 60-fold) microRNAs in diabetic cataract [177]. Significant downregulation of miR-204-5p (~ 67-fold) and miR-204-3p (> 12-fold) has also been associated with the human donor lens ‘capsular-bag’ model of PCO in vitro [176]. Experimental overexpression of miR-204-5p, by transfection of primary lens epithelial cells (LECs) from the capsular-bag PCO model, increased expression of the epithelial marker, E-cadherin, and decreased expression of the epithelial-to-mesenchymal transition (EMT) markers, alpha smooth muscle actin (α-SMA), and vimentin. Further, miR-204 overexpression was associated with enhanced repression of transforming growth factor beta-2 (TGFβ2)-induced EMT by direct targeting of the DNA-binding protein mothers against decapentaplegic homolog 4 (SMAD4) [[176]]. Similarly, in a mouse lens capsular-bag model of PCO, upregulation (> 2-fold) of miR-204 (by pre-miR-204 transfection) was associated with downregulation (15%) of *Meis*, inhibition of LEC migration and expansion, and attenuated expression of the EMT-marker, α-SMA [172]. These observations support a direct role for miR-204 in the suppression of EMT by targeting of the TGF-β/SMAD signaling pathway.

Besides EMT, miR-204 has been associated with differential regulation of oxidative stress-related genes in human age-related cataract [175]. Microarray analysis of cataractous lenses suggested that downregulation of miR-204 was associated with a predominant up-regulation of pro-oxidative genes (e.g., thioredoxin-interacting protein, *TXNIP*), whereas similar numbers of anti-oxidative genes were upregulated (e.g., glutathione peroxidase 1, *GPX1*) or downregulated (e.g., aldehyde dehydrogenase 1A3, *ADH1A3*). Remarkably, in silico analysis predicted that miR-204 not only targeted the 3′-UTR of pro-oxidative genes (e.g., *TXNIP*) for transcriptional repression but also targeted the 5′-TATA-box promoter-sequence of anti-oxidative genes (e.g., *ALDH1A3*) for transcriptional activation [175]. Thus, it was proposed that downregulation of miR-204 caused inhibition of anti-oxidative genes and activation of pro-oxidative genes suggesting that a novel miR-204-TATA box/3′-UTR gene-regulation network contributes to cataract pathogenesis [175].

In addition to age-related cataract and PCO, miR-204 was reported to be downregulated (> 4-fold) in central anterior LEC samples from young children (1–4 years) undergoing surgery for congenital (‘pulverulent’) cataract that typically presents at birth or during infancy [174]. Transfection studies of a human LEC-line (HLE-B3) and mouse capsular-bag LECs have further revealed a negative correlation between miR-204 and *Meis2* transcript levels consistent with a role for miR-204 regulation in lens development and congenital cataract pathogenesis [174].

#### TRPM3 in cataract

The first unambiguous human disease association for *TRPM3* was discovered in a 5-generation Caucasian-American family segregating pediatric cataract with autosomal dominant transmission that mapped to chromosome 9q [81]. Approximately 60% of individuals affected with cataract were also diagnosed with high-tension glaucoma (IOP > 30 mmHg) and anterior eye defects including anterior segment mesenchymal dysgenesis (ASMD), Haabs striae (horizontal breaks in Descemet’s membrane in the cornea), megalocornea, mild correctopia (pupil displacement), and persistent pupillary membrane, suggesting that TRPM3 function extends beyond the lens to the anterior segment (e.g., iris, cornea, and ciliary body). The underlying heterozygous transition (c.195A>G) located in exon-4 of *TRPM3* (RefSeq variant-9) was not present in the Exome Aggregation Consortium (ExAC) database and was predicted in silico to exert damaging effects on the function of at least one channel isoform (RefSeq isoform-k). Transfection studies of a recombinant hTRPM3-GFP reporter construct harboring the human cataract mutation have revealed that the Ile>Met substitution introduced an alternative translation start-site located 89 codons upstream from the native methionine found in at least eight other TRPM3 transcript variants and channel isoforms (Refseq variants 1–8, isoforms a-h). Thus, in addition to damaging effects on isoform-k function, the novel Ile>Met start-site may exert deleterious effects on multiple RefSeq channel isoforms by extending their N-termini with 89 novel amino-acids. Recently, a second missense mutation (c.3920G>C, p.Arg1307Thr) located in exon-29 of *TRPM3* has been discovered in a Chinese nuclear family segregating pediatric cataract [82]. This heterozygous G>C transversion was not present in the ExAC database; however, in silico analysis (Polyphen-2 and SIFT score) predicted that the non-conservative p.Arg1307Thr substitution may be functionally benign and has been designated as a variant of ‘uncertain significance’ [82].

### TRPM3_miR-204 in retinal development and disease

#### MiR-204 in retinal neurons

In medaka embryos, differential regulation of miR-204 has been directly implicated in retinal development [164, 169]. Beyond microphthalmia and lens dysgenesis, morpholino knockdown of miR-204 resulted in aberrant dorso-ventral patterning of the retina associated with failed optic fissure closure or ventral coloboma with incomplete penetrance (in 30% of small eyes) [164]. Knockdown of miR-204 resulted in upregulation of multiple putative target genes including several involved in neurogenesis and axon guidance. For example, miR-204 repression resulted in upregulation of genes coding for an ephrin ligand (ola-*Efnb3*) and an ephrin receptor (ola-*Ephb2*) that was reversed by miR-204 overexpression during retinal development in medaka embryos. Most miR-204 knockdown embryos (65%) displayed retinal ganglion cell (RGC) axon pathfinding defects that cause axons to invade other retinal layers rather than extend along the optic nerve fiber layer to vision centers in the brain. Conversely, miR-204 overexpression resulted in aberrant projection of axons to the contralateral optic nerve and ectopic rostral projection of axons to the telencephalon rather than the optic-tectum. Rescue of these RGC axon misguidance defects was achieved by co-injection of morpholinos for miR-204 and *Ephb2* or *Efnb3* suggesting that miR-204 participates in RGC axon growth and/or guidance, in part, by targeting the ephrin-B receptor signaling pathway [169].

In mouse retinal neurons, miR-204 (and miR-211) has been found to be reversibly up/downregulated during light/dark adaptation independent of the circadian clock [160]. During dark adaptation, miR-204 was downregulated and upregulated during light adaptation as a result of rapid miRNA decay and increased transcription (~ 2-fold), respectively. Retinal *Trpm3* transcript levels were also upregulated, particularly in the INL, upon light exposure. The physiological role of such light-induced regulation in the inner retina is unclear. However, in the case of miR-204, it has been speculated that high turnover facilitates assembly of new ribonucleoprotein complexes termed miRNPs to cope with transcriptional changes during neuronal activity [160].

#### MiR-204 in RPE

In human fetal (hf) RPE cells, the constitutively high miR-204 levels (~ 10,000 copies/cell) have been functionally linked with altered gene expression that maintains epithelial cell physiology [139]. First, miR-204 inhibition resulted in upregulation of target-genes involved in TGF-β signaling (*TGFBR2* and *SNAI2*) that are known to promote EMT. Second, miR-204 inhibition decreased (~ 80%) trans-epithelial electrical resistance associated with reduced claudin gene expression and tight-junction formation that is required to maintain the epithelial barrier function of the RPE. Third, miR-204 inhibition triggered apical membrane hyper-polarization resulting from ion-channel activity (e.g., K+ channel) that is required for normal RPE-photoreceptor interactions during the visual cycle. Thus, as in the lens, miR-204 participates in preservation of the epithelial cell phenotype of the RPE [139].

Prolonged culture (4 months) of confluent ARPE-19 cells resulted in massive upregulation (> 2000-fold) of miR-204 (and miR-211) that was associated with differentiation of a native RPE phenotype including a cobblestone epithelial morphology and intense pigmentation [135]. Subsequent RNA-seq and qPCR confirmed that this differentiated ARPE-19 phenotype was accompanied by RPE-specific gene expression including genes involved in melanogenesis (e.g., *MITF, TYR, TRPM1*) and 11-*cis* retinal pigment regeneration in the visual cycle (e.g., *RPE65, RDH10*) [135]. Strong upregulation of miR-204 (~ 13-fold) accompanied trans-differentiation of RPE cells from a human parthenogenetic embryo stem cell (hPESCs) line [178]. MiR-204 overexpression in transfected hPESCs resulted in decreased levels of β-catenin interacting protein 1 (CTNNBIP1)—an inhibitor of the Wnt/β-catenin signaling pathway. Reporter gene assays confirmed that CTNNBIP1 is a direct target of miR-204 further supporting a role for miR-204 in modulating Wnt/β-catenin signaling during RPE differentiation [178].

In developing mouse RPE, conditional loss of the RNase III nuclease Dicer1, which cleaves pre-miRNAs, resulted in significant depletion (~ 11-fold) of miR-204 along with upregulation of several predicted target genes including *Meis2* [162]. Dicer1-deficient RPE in vivo did not exhibit overt changes in cell morphology, identity, or fate (e.g., EMT) but, instead, was associated with increased cell density, reduced cell size, and arrested development of adjoining photoreceptors due to failed assembly of outer segment disk membranes. These data suggest that miR-204 supports RPE differentiation and maturation of adjacent photoreceptors in vivo, especially morphogenesis of the outer segments—a key structure for phototransduction [162].

Recently, mice lacking miR-204 were found to develop age-related (~ 9 months) RPE/retinal defects including hyper-autofluorescent (white) deposits, abnormal light-induced electrophysiological responses, increased microglia migration to the RPE, and impaired phagocytosis of photoreceptor outer segments accompanied by rhodopsin build-up in the RPE [179]. Further, knockdown of miR-204 in primary cultures of human RPE resulted in rhodopsin accumulation, elevated autophagy markers (e.g., p62), increased autophagic vesicles, and decreased lysosomes. Both miR-204-null mouse RPE and miR-204-knockdown human RPE also exhibited increased expression of ras-related protein Rab22a, an inhibitor of endosome maturation and a direct target of miR-204. Together, these observations implicate miR-204 in modulating the endolysosomal and/or autophagy pathways to avoid pathogenic changes in the RPE/retina that resemble those found in patients with age-related macular degeneration (AMD) [179].

#### MiR-204 in glaucoma and retinoblastoma

Downregulation of miR-204 (~ 4-fold) has been reported in a rat model of advanced glaucomatous retinal damage experimentally induced by chronic elevation of intraocular pressure [180]. In particular, miR-204 was one of eight downregulated miRNAs correlated with upregulation of several validated and predicted target genes involved in EMT and extracellular matrix (ECM) remodeling further supporting a regulatory role for miR-204 in modulating TGF-β signaling.

In a rat model of optic nerve crush injury, miR-204 upregulation in retinal blood vessels was accompanied by decreased expression of growth-associated protein-43 (GAP-43) [181]. Similarly, GAP-43 expression was decreased in rats after ocular injection of miR-204 mimic, whereas ocular injection of miR-204 inhibitor increased GAP-43 expression. Increased TUNEL-positive retinal cell death was also associated with optic nerve injury or miR-204 overexpression suggesting that miR-204 promoted apoptosis of retinal cells by inhibiting GAP-43 [181].

Downregulation of miR-204 (~ 2.5-fold) has been observed in human retinoblastoma (RB) tissues and cell lines when compared to normal pediatric retinas [182]. Restoration of miR-204 levels inhibited RB cell migration and invasion capability in vitro and decreased RB tumor growth in vivo. RB miR-204 levels were inversely correlated with those of cyclin D2 (CCND2) and matrix-metalloprotease-9 (MMP9). Both CCND2 and MMP9 transcripts harbor 3′-UTR recognition sites for miR-204 suggesting that miR-204 may play a tumor suppressor role in RB progression by targeting CCND2 and MMP9 [182].

#### MiR-204 in retinal dystrophy

In keeping with its role in vertebrate eye development, the first identified mutation in *MIR204* has been found to underlie an inherited (autosomal dominant) pediatric form of bilateral retinal (rod-cone) dystrophy and iris coloboma with or without congenital cataract (RDICC, OMIM 616722) segregating in a 5-generation British family [83]. The causative heterozygous transition (n.37C>T) was located within the phylogenetically conserved (7-nucleotide) seed-region of *MIR204* that is essential for target-transcript recognition and downregulation (Fig. 2). The n.37C>T point-mutation was not predicted in silico to destabilize precursor (pre) miR-204 secondary structure and did not significantly affect expression levels in transfected cells of either mature, processed miR-204, or premiR-204 suggesting that a loss-of-function or haplo-insufficiency mechanism was not the underlying cause of disease. Instead, functional overexpression in transfected ARPE-19 cells followed by RNA sequencing studies, revealed that the mutant miR-204 targeted multiple novel and aberrant mRNA transcripts and impaired recognition of several authentic wild-type miR-204 targets consistent with a deleterious gain-of-function mechanism. Overexpression of the n.37C>T mutant miR-204 by injection of medaka fish embryos recapitulated (~ 90% penetrance) aspects of the human eye phenotype including coloboma and increased photoreceptor (rod and cone), but not RPE, TUNEL-positive cell death. Further, morpholino knockdown of miR-204 in medaka retina resulted in a significant reduction of the flash ERG b-wave amplitude recorded under dim-light or scotopic (rod-photoreceptor dominant) conditions. Combined these observations suggest that, in addition to the adjacent RPE, miR-204 plays an important role in photoreceptor differentiation, function, and survival in the outer retina [83].

#### TRPM3 in retina

Visual function testing has shown that loss of *Trpm3* function in the mouse retina results in a relatively normal ERG with negligible effects on either dim-light (scotopic) or bright-light (photopic) ERG a-waves (photoreceptor-derived) and ERG b-waves (OPL-derived Muller and ON-bipolar cells) [153]. Thus, in contrast to TRPM1, TRPM3 appears to play a minimal role in visual processing by the ON-bipolar cell pathway (IPL inner sub-lamina b activity) that is activated (depolarized) by light exposure. However, TRPM3 may function in regulation of the OFF-bipolar cell pathway (IPL outer sub-lamina a activity), including OFF ganglion cells accounting for ~ 40% of the GCL, that is inhibited (hyperpolarized) by light exposure [153].

In contrast to *Trpm1*-null mice, which exhibited a profound deficit in pupillary light reflex (PLR), *Trpm3*-null mice displayed a more subtle attenuated PLR under both bright light (rod/cone/melanopsin-response) and dim light (rod/cone-response) conditions, consistent with a role in non-image photoreception [93]. While *Trpm3*-null mice exhibited rapid pupil constriction in response to bright light that was maintained during illumination they failed to achieve full pupil constriction (i.e., ~ 80% of wild-type). They also displayed an abnormal post-illumination pupil response with a more rapid pupil dilation compared to the sustained post-stimulus pupil constriction of wild-type. In response to dim light, pupil constriction in *Trpm3*-null mice was approximately 45% of that in wild-type. Muscarinic stimulation of the eye by topical administration of the cholinergic agonist, carbachol, resulted in complete pupil constriction suggesting that the ciliary and iris-sphincter muscles were not functionally impaired in *Trpm3*-null mice. Since TRPM3 was not detected in outer retina photoreceptors (rods and cones) or photosensitive (melanopsin-expressing) retinal ganglion cells (pRGCs) of the inner retina, it was proposed to play a more distal role in regulating pupillary responses to light that may involve retinal Muller glial cells and the ciliary body where TRPM3 was enriched [93].

In human RPE cells, the apical membrane co-localization of TRPM3 and tight-junctions (ZO1) may modulate junctional permeability and barrier function, whereas TRPM3 enrichment at the base of the primary cilium may contribute to sensing light-induced Ca^2+^ concentration changes in the sub-retinal space (or inter-photoreceptor matrix) between the RPE and photoreceptors during the visual cycle [136]. Prolonged culture of human ARPE-19 cells (4 months) resulted in upregulation (3–4-fold) of TRPM3 (and TRPM1) transcripts to levels approaching those of native human RPE cells [135]. However, exposure of cultured ARPE-19 cells to pro-inflammatory cytokines (e.g., IFN-y) resulted in downregulation of TRPM3 (3–4-fold) and miR-204 (15–95%) transcripts along with other genes indispensable for RPE function, including *MITF* and *TRPM1_MIR211* [183]. Since inflammation is believed to exacerbate AMD pathogenesis, release of pro-inflammatory cytokines from immune cells infiltrating the posterior eye may trigger the RPE dysfunction implicated in AMD [183].

### TRPM3_miR-204 in cornea, trabecular meshwork, and optic nerve

#### MiR-204 in corneal wound healing

Dramatic downregulation (> 200-fold) of miR-204 has been detected during corneal wound healing following traumatic corneal epithelial injury (by cell scraping) in mice [158]. Conversely, overexpression of miR-204 in transfected human corneal epithelial cells (HCECs) inhibited cell proliferation and migration by inducing cell cycle G1-arrest. These findings suggest that miR-204 downregulation promotes corneal epithelial wound healing following injury and that miR-204 can be considered as a negative biomarker for corneal wound healing response [158]. In broad support of this premise, miR-204-5p was markedly upregulated (~ 5-fold) in corneal epithelia of type-1 diabetic (*Ins2*^*Akita/+*^) mice [184]. This miR-204 upregulation was correlated with downregulation of the longevity gene coding for the NAD-dependent protein deacetylase sirtuin-1 (SIRT1)—a confirmed direct target for miR-204 [158, 184]. Under high glucose conditions, miR-204 was increased in a mouse corneal/limbal epithelium (TKE2) cell line, whereas SIRT1 and the cell-cycle related gene coding for cyclin D1 (CCND1) were downregulated inducing cell-cycle (S-phase) arrest. However, antagomiR inhibition of miR-204 expression in hyperglycemic TKE2 cells resulted in upregulation of SIRT1 and CCND1 expression accompanied by increased cell growth and restored cell cycle progression. Further, sub-conjunctival injection of miR-204 antagomiR promoted corneal wound healing response in diabetic mice following corneal epithelial injury. These data suggest that miR-204 exerts a negative effect on corneal wound healing in diabetic keratopathy by targeting SIRT1 thereby contributing to delayed progression of the epithelial cell cycle [184].

#### MiR-204 in corneal neovascularization

Loss of miR-204 expression (~ 20-fold) has been reported in mice lacking Kelch-like Ect2-interacting protein (KLEIP)—a genetic model of spontaneous corneal neovascular dystrophy [185]. Such miR-204 downregulation in late-stage *Kleip*-null corneas was correlated with strong upregulation of the proangiogenic factor angiopoietin-1 (ANGPT1) and its receptor tyrosine kinase (TIE2), but not with canonical vascular endothelial growth factors A-C (VEGFA-C). Bioinformatics analysis identified an miR-204 binding site in the ANGPT1 transcript and overexpression of miR-204 mimic in transfected vascular endothelial cells resulted in downregulation of ANGPT1 consistent with the latter acting as a miR-204 target-gene and supporting a role for the miR-204-*Angpt1* pathway in *Kleip*-null corneal neovascular dystrophy [185]. In addition, mice undergoing suture-induced corneal neovascularization exhibited miR-204 downregulation (~ 4-fold) that correlated with the vascularized area of corneal epithelium [186]. Conversely, sub-conjunctival injection of miR-204 agomiR inhibited neovascularization after corneal injury and decreased expression of VEGFA and that of its receptor VEGFR2. Overexpression of miR-204 agomiR also inhibited VEGFA upregulation in primary corneal (limbal) epithelial cells undergoing biomechanical stress in vitro and suppressed proliferation, migration, and tube-formation in microvascular endothelial cell cultures—consistent with a role in inhibition of corneal neovascularization [186]. Similarly, mice subject to alkali-induced corneal neovascularization exhibited downregulation of miR-204 (~ 10-fold) along with the simultaneous upregulation of over 200 corneal genes predicted to be targets for miR-204 [187]. These upregulated genes included several vasculogenic genes including that for ANGPT1, which can activate the phosphatidylinositol-3-kinase/AKT-serine/threonine kinase 1 (PI3K/AKT1) pathway along with one of its down-stream targets VEGF. Corneal delivery of miR-204, by recombinant adeno-associated virus (rAAV) vector, normalized the expression of multiple predicted target genes and pathways that were upregulated by alkali-induced corneal injury [187]. Combined, these observations suggest that miR-204 acts as an endogenous suppressor of corneal neovascularization and represents a potential therapeutic target for inhibiting corneal angiogenesis.

#### MiR-204 in trabecular meshwork

Microarray and qPCR analyses have associated downregulation of miR-204 (~ 2.5-fold) with increased senescence in primary cultures of human trabecular meshwork (HTM) cells [141, 142]. Similar studies have revealed that miR-204 overexpression in transfected primary HTM cells resulted in downregulation (> 1.5-fold) of at least 12 genes with predicted and/or validated 3′-UTR miR-204 binding-sites [143]. These downregulated miR-204 target genes included inhibitors of apoptosis (e.g., *BCL2L2*, *BIRC2*), activators of the endoplasmic reticulum (ER)-stress response (e.g., *HSPA5*/*BiP*, *DDIT3/CHOP*), and mediators of the inflammatory response (e.g., *IL8, IL11*). When subject to oxidative-stress (H_2_O_2_) or ER-stress (tunicamycin), HTM cells overexpressing miR-204-mimic displayed increased apoptotic cell death, accumulation of oxidized protein (carbonylation) along with decreased expression of ER-stress markers, and inflammatory factors [143]. Other miR-204 target genes downregulated in HTM cells overexpressing miR-204 mimic include the fork-head box C1 transcription factor (*FOXC1*)—a causative gene for anterior segment dysgenesis known as Axenfeld-Rieger syndrome with a ~ 50% risk for high tension glaucoma—along with several downstream *FOXC1*-target genes, notably *MEIS2* [188]. Further, transfection studies have shown that miR-204 mimic inhibited serum-induced contraction of HTM cells cultured within collagen gels suggesting that miR-204 participates in regulating trabecular meshwork contractibility to modulate aqueous humor drainage and intraocular pressure (IOP) in vivo [189]. Overall, these data support a multi-functional role for miR-204 in HTM cells and, more broadly, in anterior eye development and disease.

#### TRPM3 in optic nerve

TRPM3 channels have been shown to participate in replenishment of ER Ca^2+^ stores by store-operated calcium entry (SOCE) and in sustainability of ATP-mediated Ca^2+^ signaling in white matter glial cells (astrocytes and oligodendrocytes) derived from mouse optic nerve [154].

## Conclusions

**Fig. 5:**
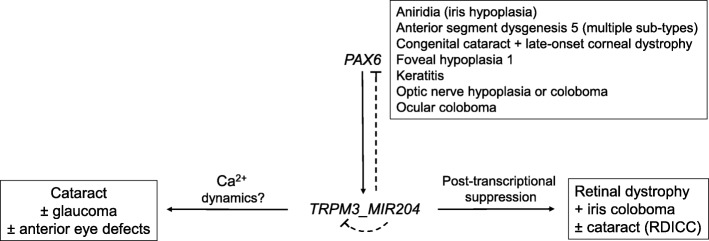
Schematic of *PAX6* and *TRPM3_MIR204* in human inherited eye disease

From ‘light-blindness’ in fruit flies (trp) to ‘night-blindness’ in humans (TRPM1), TRP channels have been identified as evolutionarily important Ca^2+^ sensors in the photosensitive retina [11, 36]. Recently, the TRPM3_miR-204 locus has emerged as an important transcriptional target for PAX6 that serves to highlight the complex convergence of TRP channels and micro-RNAs with mammalian eye development and disease [165]. In humans, mutation of *TRPM3* underlies pediatric cataract with or without glaucoma and anterior segment defects, whereas mutation of *MIR204* underlies retinal dystrophy and iris coloboma with or without cataract [81, 83]. Notably, *PAX6* mutations in humans also underlie a variable pan-ocular phenotype(s) including aniridia (iris hypoplasia), foveal hypoplasia, anterior segment dysgenesis 5 (ASD5), late-onset corneal dystrophy, ocular coloboma, and congenital cataract [190, 191]. Such overlap in eye disease phenotypes supports a functional synergy between PAX6, TRPM3, and miR-204 during eye development (Fig. 5). Beyond genetic mutations, however, much remains to be learned about the cellular and molecular mechanisms underlying TRPM3_miR-204 function and dysfunction in ocular gene expression and calcium dynamics.

### MiR-204 and ocular gene expression

Mir-204 expression has been prominently associated with development and differentiation of multiple eye tissues. In the retina, differential regulation of miR-204 has been linked with development of the neural retina that mediates phototransduction, differentiation of the RPE that supports adjacent photoreceptor function and forms the outer blood-retinal barrier of the eye, and with retinal disease. First, miR-204 has been implicated in RGC axon guidance to the visual cortex, in part, by targeting the *Efnb2*-*Ephb2* pathway [164, 169]. Second, high levels of miR-204 has been shown to preserve epithelial differentiation and barrier function of the RPE to support photoreceptor function by targeting genes involved in EMT (e.g., *TGFBR2*) and Wnt/β-catenin signaling (e.g., *CTNNBIP1*) [139, 178, 162]. Third, light-regulated changes in miR-204 transcription and turnover may aid assembly of new miRNP complexes required for transcriptional changes during neuronal activity in the inner retina [160]. Fourth, lack of miR-204 resulted in an age-related RPE/retinal (AMD-like) phenotype associated with elevated Rab22a-mediated inhibition of the endolysosome/autophagy pathways [179]. Fifth, mutation of miR-204 seed-region causes rod-cone dystrophy associated with altered target gene expression suggesting a key role for miR-204 in photoreceptor function and survival in the outer retina [83]. Sixth, miR-204 downregulation in glaucomatous retina was associated with upregulation of genes involved in EMT and ECM-remodeling suggesting that miR-204 may modulate TGFβ-signaling [180, 181]. Seventh, miR-204 downregulation in retinoblastoma suggested that it may act as a tumor suppressor by targeting CCND2 and MMP9 [182].

Differential regulation of miR-204 expression has been directly associated with development and differentiation of the crystalline lens that facilitates fine-focusing of images onto the retina and with clinically distinct types of cataract formation. First, miR-204 controls lens morphogenesis, in part, by targeting *Meis2* and *Ankrd13a* [164, 170]. Second, miR-204 upregulation by *Pax6* drives lens epithelial cell fate determination by targeting genes involved in neurogenesis (e.g., *Sox11*) and cell motility (e.g., *Myo10*) [165]. Third, miR-204 downregulation in age-related cataract and PCO suggest that miR-204 targets genes involved in EMT (e.g., αSMA) and oxidative stress (e.g., *ALDH1A3*), whereas miR-204 downregulation in congenital cataract implicated targeting of *Meis2* [172–176].

Beyond retina and lens, differential regulation of miR-204 has been implicated in pathophysiology of the cornea that generates most of the eye’s refractive/focusing power and the trabecular meshwork that drains aqueous humor outflow from the anterior eye. First, miR-204 downregulation promoted corneal epithelial wound healing, whereas miR-204 upregulation inhibited wound healing in diabetic keratopathy by targeting SIRT1 and CCND1 to inhibit the cell cycle [158, 184]. Second, miR-204 downregulation was associated with corneal neovascularization after injury, whereas miR-204 upregulation inhibited neovascularization by targeting genes involved in vasculature formation (e.g., *Angpt1*) [185–187]. Third, in trabecular meshwork cells, miR-204 downregulation was associated with cellular aging, whereas miR-204 upregulation targeted multiple genes involved in apoptosis (BCL2L2), ER-stress (DDIT3/CHOP), inflammatory response (IL8), and anterior eye morphogenesis (*FOXC1*) [141–143, 188]. Fourth, miR-204 upregulation inhibited the contractile ability of trabecular meshwork cells that is believed to modulate aqueous fluid drainage and IOP [189].

Clearly, miR-204 plays complex multifunctional roles in regulating ocular gene expression. While there is some overlap of miR-204 target genes/pathways in different eye tissues (e.g., EMT in lens and RPE), so far most miR-204 targets appear to be cell and/or disease context dependent. However, despite its multiple target genes, functional loss of miR-204 does not appear to negatively impact eye development and differentiation in mice that eventually acquire (~ 9 months) an AMD-like phenotype [179]. Such sparing of eye development raises the possibility of functional redundancy or compensation between miR-204 and miR-211, which share the same seed-region sequence and predicted target genes, and provides a rationale for investigating the ocular phenotype of miR-204 and/or miR-211 null mice.

### TRPM3 and ocular Ca^2+^ dynamics

TRPM3 is widely expressed in eye tissues and has been tentatively implicated in both neuronal and epithelial cell Ca^2+^ dynamics. In retinal neurons, TRPM3 has been proposed to act as a Ca^2+^ sensor in the OFF-pathway of bipolar cells and a subset of ganglion cells [153]. Similarly in RPE, TRPM3 has been speculated to modulate tight-junction permeability and to function as a Ca^2+^ sensor for the sub-retinal space between the RPE and photoreceptors during the visual pigment cycle [136]. TRPM3 channels may also contribute to Ca^2+^ homeostasis (SOCE) in optic nerve glial cells [154]. Functional loss of TRPM3 caused a mild deficit in pupil (iris) constriction in response to light that may involve retinal Muller glia and/or ciliary body dysfunction [93].

In the lens, loss of TRPM3 has not been associated with a cataract phenotype in mice, raising the possibility of functional redundancy or compensation by other lens TRP channels. For example, TRPV1 and TRPV4 have been shown to participate in maintaining an intracellular hydrostatic pressure gradient within the lens [192]. By contrast, mutation of TRPM3 in humans underlies pediatric cataract (with or without glaucoma and anterior segment defects) suggesting a deleterious gain-of-function mechanism that may compromise lens Ca^2+^ homeostasis [81]. Elevated cytoplasmic Ca^2+^—believed to result from activation of a non-selective cation conductance—has long been implicated in the pathophysiology of lens aging and cataract formation in humans and experimental animals [193]. Besides pediatric cataract, TRPM3 channels may also contribute to the historically enigmatic non-selective cation current associated with age-related cataract. Moreover, based on extraocular TRPM3 channel functions including secretion by pancreatic β cells [40], mechano-sensing by vascular smooth muscle cells [51] and osmo-sensing by renal epithelial cells [55], it is conceivable that TRPM3 channels participate in ocular epithelial cell volume regulation and/or ion and fluid transport including aqueous humor secretion by the ciliary epithelium that maintains IOP. Further functional expression and disease-modeling studies (e.g., CRISPR-Cas9 gene-editing) will be required to elucidate the pathogenic effects of known and newfound TRPM3 mutations on Ca^2+^ dynamics in the lens and other anterior eye tissues including the ciliary body and iris.

In addition to cellular aspects of TRPM3 channel function and dysfunction in the eye, several molecular aspects warrant further investigation. First, since alternative splicing can alter the functional properties of TRPM3 channels [110], the expression profile (i.e., exon usage) of TRPM3 transcript variants and the functional properties of corresponding TRPM3 isoforms in ocular tissues requires further studies. In particular, how do short TRPM3 isoforms that lack transmembrane channel domains serve to regulate full-length TRPM3 channels as proposed for short and long TRPM1 channels [113]? Alternatively, the short transcript variants of TRPM3 that all start upstream (5′-) and end downstream (3′-) from miR-204 may facilitate a transcriptional mechanism to independently regulate miR-204 and TRPM3 expression during eye development. Further molecular investigations include characterization of ligand-binding and heat-sensing domains in TRPM3 channels. Since PS is unlikely to cross the plasma membrane due to its negatively charged sulfate side-group, it is believed to bind a conserved stereo-specific protein binding-site that likely resides within the four extracellular-transmembrane regions of TRPM3 channels [40, 47, 60, 61, 194]. By contrast, CIM0216 is membrane permeable and activates TRPM3 channels in a membrane-delimited manner acting at sites both outside and inside the membrane [42, 114]. In the case of heat-sensing domains, it is notable that PS-activation of a truncated hTRPM3 channel (hTRPM3_1325_) was not enhanced by heat [68] raising the possibility that C-terminal sequences may be involved in heat sensitivity. Other cytoplasmic sequences including those at the C-terminus may also serve as interaction sites for TRPM3 channel inhibition by Gβγ sub-units [76–79].

Second, the true physiological agonists and antagonists for TRPM3 channels in the eye remain elusive. The endogenous neurosteroid, PS, is the most widely used experimental agonist and it activates TRPM3 channels within physiological concentration and temperature ranges suggesting that integration of chemical and thermal stimuli to activate cation conductance may be physiologically and/or pharmacologically relevant. Since heat (37 °C) sensitizes TRPM3 channels to activation by PS at levels close to those in blood (1–5 μM range at birth, 100–150 nM range in adults), such chemical-thermal synergy raises the possibility that circulating PS may act as an authentic agonist for TRPM3 channels at body core temperature [57]. However, since TRPM3 channels are constitutively active (i.e., open), it is currently unclear whether PS and other experimental agonists (e.g., CIM0216) actively open or gate the channels or simply modulate the open-probability of already gated channels [58]. Intriguingly, PS has been implicated in decreasing IOP and to be neuroprotective in a rat model of glaucoma [195]. These observations raise the possibility that TRPM3 channels may contribute to IOP homeostasis. Elevated IOP is a major risk factor for glaucoma in humans and the majority of family members segregating a cataract-causing mutation in *TRPM3* also developed high-tension glaucoma [81]. Several experimental inhibitors of TRPM3 channels exhibit strong anti-nociceptive properties (e.g., NSAIDs)—suggesting a role for TRPM3 channels in ocular pain—while others are dietary compounds (e.g., citrus flavanones) [71–74]. TRPM3 channel inhibition appears to be well tolerated, at least in *Trpm3*-null mice, but may cause unwanted side effects in humans including reduced insulin secretion and/or noxious heat insensitivity. However, none of the known TRPM3 channel agonists or antagonists are likely to be specific enough for pharmacologic studies in vivo [58].

Finally, aside from physiological or pharmacological activation and inhibition, the constitutive intracellular regulation and downstream signaling mechanisms of TRPM3 channels in ocular tissues remain to be elucidated. Using transfected (HEK293) cells, at least three intracellular mechanisms are believed to regulate TRPM3 channels. CaM binding and PIP hydrolysis serve as activators, whereas Gβγ-subunits act as inhibitors [66, 79, 101]. Ca^2+^ influx from TRPM3 channels triggers a signaling cascade of MAPKs (e.g., ERK1/2) and stimulus response transcription factors (e.g., AP-1) that in turn alter expression of delayed response genes [123]. Although HEK293 cells are derived from primary embryonic human kidney and display an epithelial morphology, it is unclear that the same signaling pathways triggered by TRPM3 channels will be utilized in both ocular epithelia and ocular neurons. Further, the identities of delayed response genes targeted by TRPM3 channel signaling in ocular epithelia and neurons remain to be discovered.

Future multidisciplinary studies including genomics, transcriptomics, proteomics, and metabolomics of the TRPM3_miR-204 locus will likely provide new insights regarding ocular health and disease. Regardless of its precise ocular function(s) however, the TRPM3 gene along with that for miR-204 appear to have co-evolved as a target locus for PAX6 to coordinate regulation of gene expression with Ca^2+^ dynamics during vertebrate eye development.

## Supplementary information

**Additional file 1.Table S1.** Schematic summary of human TRPM3 transcript variants and protein isoforms. (A) RefSeq variants (1–23) and isoforms (a-w). (B) Predicted variants and isoforms (X1–8, X10–13, X15, X18–19). Gray boxes denote exons included in each variant and numbers denote amino-acid (AA) counts for each isoform. Asterisks indicate translation stop codons. NT, nucleotide. AA, amino acid

**Additional file 2.Table S2.** Schematic summary of mouse TRPM3 transcript variants and protein isoforms (A) RefSeq variants (1–30) and isoforms (a-z, aa-dd). (B) Predicted variants and isoforms (X1–15, X18–21). Gray boxes denote exons included in each variant and numbers denote amino acid counts for each isoform. Asterisks indicate translation stop codons

**Additional file 3. Table S3.** Ocular expression profile of the TRPM3 and miR-204 genes in humans (*TRPM3*, *MIR204*) and mice (*Trpm3*, *Mir204*)
