# Supplementary material for: TRPM3_miR-204: a complex locus for eye development and disease
Source: Hum Genomics. 2020 Feb 18;14:7. doi: 10.1186/s40246-020-00258-4 (PMC7027284; doi:10.1186/s40246-020-00258-4)
Supplement: Supplementary file 1 — Additional file 1.Table S1. Schematic summary of human TRPM3 transcript variants and protein isoforms. (A) RefSeq variants (1–23) and isoforms (a-w). (B) Predicted variants and isoforms (X1–8, X10–13, X15, X18–19). Gray boxes denote exons included in each variant and numbers denote amino-acid (AA) counts for each isoform. Asterisks indicate translation stop codons. NT, nucleotide. AA, amino acid [file 40246_2020_258_MOESM1_ESM.pdf]

Supplementary Table 1. Human *TRPM3* transcript variants and protein isoforms.

Variant (ID number)	NT count	Isoform (ID number)	AA count	Ex1	Ex2	Ex3	Ex4	Ex5	Ex6	Ex7	Ex8	Ex9	IVS9	Ex10	Ex11	Ex11a	Ex12	Ex13	Ex14	Ex15	Ex16	Ex17	Ex18	Ex19	Ex20	Ex21	Ex22	Ex23	Ex24	Ex25	Ex26	Ex27	Ex28	Ex29	Ex30
A. RefSeq (GRCh38.p13)																																			
1 (NM_020952.4)	5940	a (NP_066003)	1554					1	73	114	172		*MIR204*	230	271		296	329	374	391		438	448	545	621	677	724	767	851	910	960	1027	1071	1554*	
2 (NM_024971.5)	5976	b (NP_079247)	1566					1	73	114	172		*MIR204*	230	271		296	329	374	391	403	450	460	557	633	689	736	779	863	922	972	1039	1083	1566*	
3 (NM_206944.3)	5910	d (NP_996827)	1544					1	73	114	172		*MIR204*	230	271		296	329	374	391		438		535	611	667	714	757	841	900	950	1017	1061	1544*	
4 (NM_206945.3)	5946	e (NP_996828)	1556					1	73	114	172		*MIR204*	230	271		296	329	374	391	403	450		547	623	679	726	769	853	912	962	1029	1073	1556*	
5 (NM_206946.3)	6015	f (NP_996829)	1579					1	73	114	172	197	*MIR204*	255	296		321	354	399	416		463	473	570	646	702	749	792	876	935	985	1052	1096	1579*	
6 (NM_206947.3)	5985	g (NP_996830)	1569					1	73	114	172	197	*MIR204*	255	296		321	354	399	416		463		560	636	692	739	782	866	925	975	1042	1086	1569*	
7 (NM_206948.2)	1303	h (NP_996831)	230					1	73	114	172		*MIR204*	230*																					
8 (NM_001007470.1)	1378	c (NP_001007471)	255					1	73	114	172	197	*MIR204*	255*																					
9 (NM_001007471.2)	6288	k (NP_001007472)	1707		59		86	154	226	267	325		*MIR204*	383	424		449	482	527	544		591	601	698	774	830	877	920	1004	1063	1113	1180	1224	1707*	
10 (NM_001366141.1)	12158	i (NP_001353070)	1709	61			88	156	228	269	327		*MIR204*	385	426		451	484	529	546		593	603	700	776	832	879	922	1006	1065	1115	1182	1226	1709*	
11 (NM_001366142.1)	10524	j (NP_001353071)	1339	61			88	156	228	269	327		*MIR204*	385	426		451	484	529	546	558	605	615	712	788	844	891	934	1018	1077	1127	1194	1238	1333	1339*
12 (NM_001366143.1)	10488	w (NP_001353072)	1327	61			88	156	228	269	327		*MIR204*	385	426		451	484	529	546		593	603	700	776	832	879	922	1006	1065	1115	1182	1226	1321	1327*
13 (NM_001366144.1)	1538	l (NP_001353073)	385	61			88	156	228	269	327		*MIR204*	385*																					
14 (NM_001366145.1)	12319	m (NP_001353074)	1719		59		86	154	226	267	325		*MIR204*	383	424		449	482	527	544	556	603	613	710	786	842	889	932	1016	1075	1125	1192	1236	1719*	
15 (NM_001366146.1)	10649	n (NP_001353075)	1337		59		86	154	226	267	325		*MIR204*	383	424		449	482	527	544	556	603	613	710	786	842	889	932	1016	1075	1125	1192	1236	1331	1337*
16 (NM_001366147.1)	12394	o (NP_001353076)	1744		59		86	154	226	267	325	350	*MIR204*	408	449		474	507	552	569	581	628	638	735	811	867	914	957	1041	1100	1150	1217	1261	1744*	
17 (NM_001366148.1)	10694	p (NP_001353077)	1352		59		86	154	226	267	325	350	*MIR204*	410	449		474	507	552	569	581	628		725	801	857	904	937	1031	1090	1140	1207	1251	1346	1352*
18 (NM_001366149.1)	12289	q (NP_001353078)	1709		59		86	154	226	267	325		*MIR204*	383	424		449	482	527	544	556	603		700	776	832	879	922	1006	1065	1115	1182	1226	1709*	
19 (NM_001366150.1)	10583	r (NP_001353079)	1315		59		86	154	226	267	325		*MIR204*	383	424		449	482	527	544		591		688	764	820	867	910	994	1053	1103	1170	1214	1309	1315*
20 (NM_001366151.1)	10613	s (NP_001353080)	1325		59		86	154	226	267	325		*MIR204*	383	424		449	482	527	544		591	601	698	774	830	877	920	1004	1063	1113	1180	1224	1319	1325*
21 (NM_001366152.1)	10724	t (NP_001353081)	1362		59		86	154	226	267	325	350	*MIR204*	410	449		474	507	552	569	581	628	638	735	811	867	914	947	1041	1100	1150	1217	1261	1356	1362*
22 (NM_001366153.1)	1663	u (NP_001353082)	383		59		86	154	226	267	325		*MIR204*	383*																					
23 (NM_001366154.1)	10272	v (NP_001353083)	1184					1	73	114	172		*MIR204*	230	271		296	329	374	391	403	450	460	557	633	689	736	779	863	922	972	1039	1083	1178	1184*
B. Predicted (GRCh38.p13)																																			
X1 (XM_017015156)	11544	X1 (XP_016870645)	1290										*MIR204*				20	53	98	115	127	174	184	281	357	413	460	503	587	646	696	763	807	1290*	
X2 (XM_024447681)	11514	X2 (XP_024303449)	1280										*MIR204*				20	53	98	115	127	174		271	347	403	450	493	577	636	686	753	797	1280*	
X3 (XM_024447682)	11508	X3 (XP_024303450)	1278										*MIR204*				20	53	98	115		162	172	269	345	401	448	491	575	634	684	751	795	1278*	
X4 (XM_024447683)	11478	X4 (XP_024303451)	1268										*MIR204*				20	53	98	115		162		259	335	391	438	481	565	624	674	741	785	1268*	
X5 (XM_024447684)	9874	X5 (XP_024303452)	908										*MIR204*				20	53	98	115	127	174	184	281	357	413	460	503	587	646	696	763	807	902	908*
X6 (XM_024447685)	9844	X6 (XP_024303453)	898										*MIR204*				20	53	98	115	127	174		271	347	403	450	493	577	636	686	753	797	892	898*
X7 (XM_024447686)	9838	X7 (XP_024303454)	896										*MIR204*				20	53	98	115		162	172	269	345	401	448	491	575	634	684	751	795	890	896*
X8 (XM_024447687)	1589	X8 (XP_024303455)	410	61			88	156	228	269	327	352	*MIR204*	410*																					
X10 (XM_011519037)	12351	X10 (XP_011517339)	1734		59		86	154	226	267	325	350	*MIR204*	408	449		474	507	552	569	581	628		725	801	857	904	947	1031	1090	1140	1207	1251	1734*	
X11 (XM_011519038)	12344	X11 (XP_011517340)	1732		59		86	154	226	267	325	350	*MIR204*	408	449		474	507	552	569		616	626	723	799	855	902	945	1029	1088	1138	1205	1249	1732*	
X12 (XM_011519039)	12314	X12 (XP_011517341)	1722		59		86	154	226	267	325	350	*MIR204*	408	449		474	507	552	569		616		713	789	845	892	935	1019	1078	1128	1195	1239	1722*	
X13 (XM_011519040)	12145	X13 (XP_011517342)	1721	61			88	156	228	269	327		*MIR204*	385	426		451	484	529	546	558	605	615	712	788	844	891	934	1018	1077	1127	1194	1238	1721*	
X15 (XM_011519042)	12115	X15 (XP_011517344)	1711	61			88	156	228	269	327		*MIR204*	385	426		451	484	529	546	558	605		702	778	834	881	924	1008	1067	1117	1184	1228	1711*	
X18 (XM_011519045)	12078	X18 (XP_011517347)	1699	61			88	156	228	269	327		*MIR204*	385	426		451	484	529	546		593		690	766	822	869	912	996	1055	1105	1172	1216	1699*	
X19 (XM_011519046)	12239	X19 (XP_011517348)	1697		59		86	154	226	267	325		*MIR204*	383	424		449	482	527	544		591		688	764	820	867	910	994	1053	1103	1170	1214	1697*	
