# Supplementary material for: TRPM3_miR-204: a complex locus for eye development and disease
Source: Hum Genomics. 2020 Feb 18;14:7. doi: 10.1186/s40246-020-00258-4 (PMC7027284; doi:10.1186/s40246-020-00258-4)
Supplement: Supplementary file 2 — Additional file 2.Table S2. Schematic summary of mouse TRPM3 transcript variants and protein isoforms (A) RefSeq variants (1–30) and isoforms (a-z, aa-dd). (B) Predicted variants and isoforms (X1–15, X18–21). Gray boxes denote exons included in each variant and numbers denote amino acid counts for each isoform. Asterisks indicate translation stop codons [file 40246_2020_258_MOESM2_ESM.pdf]

Supplementary Table 2. Mouse *Trpm3* transcript variants and protein isoforms

Variant (ID number)	NT count	Isoform (ID number)	AA count	Ex1	Ex2	Ex3	Ex3a	Ex4	Ex5	Ex6	Ex7	Ex8	Ex9	IVS9	Ex10	Ex11	Ex12	Ex13	Ex14	Ex15	Ex16	Ex17	Ex18	Ex19	Ex20	Ex21	Ex22	Ex23	Ex24	Ex25	Ex26	Ex27	Ex28	Ex29	Ex30	Ex31	Ex32	Alias
A. RefSeq (GRCm38.p6 C57BL/6J)																																						
1 (NM_001035239.2)	6742	a (NP_001030316.1)	1721	61				88	156	228	269	327		*Mir204*	385		426	451	484	529	546		593	603	700	776	832	879	922		1006	1077	1127	1194	1238	1721*		α1
2 (NM_001035240.2)	6742	b (NP_001030317.1)	1721	61				88	156	228	269	327		*Mir204*	385		426	451	484	529	546	558	605	615	712	788	844	891	934		1018	1077	1127	1194	1238	1721*		α3
3 (NM_001035241.2)	6712	c (NP_001030318.1)	1711	61				88	156	228	269	327		*Mir204*	385		426	451	484	529	546	558	605		702	778	834	881	924		1008	1067	1117	1184	1228	1711*		α4
4 (NM_001035242.1)	6706	d (NP_001030319.1)	1709	61				88	156	228	269	327		*Mir204*	385		426	451	484	529	546		593	603	700	776	832	879	922		1006	1065	1115	1182	1226	1709*		α2
5 (NM_001035243.2)	6676	e (NP_001030320.1)	1699	61				88	156	228	269	327		*Mir204*	385		426	451	484	529	546		593		690	766	822	869	912		996	1055	1105	1172	1216	1699*		α5
6 (NM_177341.4)	5157	f (NP_796315.2)	1337		59			86	154	226	267	325		*Mir204*	383		424	449	482	527	544	556	603	613	710	786	842	889	932		1016	1075	1125	1192	1236	1331	1337*	
7 (NM_001035244.1)	3653	g (NP_001030321.1)	958						1	73	114	172	197	*Mir204*	255		296	321	354	381	398		445		542	618	674	721	764		848	958*						
8 (NM_001035245.1)	1626	h (NP_001030322.1)	234						1	73	114	172		*Mir204*	230	234*																						
9 (NM_001035246.1)	3851	i (NP_001030323.1)	255						1	73	114	172	197	*Mir204*	255*																							
10 (NM_001362487.1)	12412	j (NP_001349416.1)	1744		59			86	154	226	267	325	350	*Mir204*	408		449	474	507	552	569	581	628	638	735	811	867	914	957		1041	1100	1150	1217	1261	1744*		β10
11 (NM_001362488.1)	12382	k (NP_001349417.1)	1734		59			86	154	226	267	325	350	*Mir204*	408		449	474	507	552	569	581	628		725	801	857	904	947		1031	1090	1140	1207	1251	1734*		β11
12 (NM_001362489.1)	12376	l (NP_001349418.1)	1732		59			86	154	226	267	325	350	*Mir204*	408		449	474	507	552	569		616	626	723	799	855	902	945		1029	1088	1138	1205	1249	1732*		β6
13 (NM_001362490.1)	12373	m (NP_001349419.1)	1731		59			86	154	226	267	325		*Mir204*	383		424	449	482	527	544	556	603	613	710	786	842	889	932		1016	1087	1137	1204	1248	1731*		β17
14 (NM_001362491.1)	12337	n (NP_001349420.1)	1719		59			86	154	226	267	325		*Mir204*	383		424	449	482	527	544	556	603	613	710	786	842	889	932		1016	1075	1125	1192	1236	1719*		β3
15 (NM_001362496.1)	12375	o (NP_001349425.1)	1579						1	73	114	172	197	*Mir204*	255		296	321	354	399	416		463	473	570	646	702	749	792		876	935	985	1052	1096	1579*		
16 (NM_001362497.1)	12299	p (NP_001349426.1)	912											*Mir204*													35	82	125		209	268	318	385	429	912*		
17 (NM_001362498.1)	12283	q (NP_001349427.1)	1701		59			86	154	226	267	325		*Mir204*	383		424	449	482	509	526	548	585	595	692	768	824	871	914		998	1057	1107	1174	1218	1701*		β8
18 (NM_001362499.1)	12328	r (NP_001349428.1)	1716		59			86	154	226	267	325	350	*Mir204*	408		449	474	507	534	551	563	610		707	783	839	886	929		1013	1072	1122	1189	1233	1716*		β13
19 (NM_001632500.1)	12256	s (NP_001349429.1)	1692		59			86	154	226	267	325		*Mir204*	383		424	474	507	537	544	556	603	613	710	786	815	862	905		989	1048	1098	1165	1209	1692*		β14
20 (NM_001362501.1)	12346	t (NP_001349430.1)	1722		59			86	154	226	267	325	350	*Mir204*	408		449	474	507	552	569		616		713	789	845	892	935		1019	1078	1128	1195	1239	1722*		β12
21 (NM_001362502.1)	12337	u (NP_001349431.1)	1719		59			86	154	226	267	325		*Mir204*	383		424	449	482	527	544		591	601	698	774	830	877	920		1004	1075	1125	1192	1236	1719*		β1
22 (NM_001362503.1)	12307	v (NP_001349432.1)	1709		59			86	154	226	267	325		*Mir204*	383		424	449	482	527	544	556	603		700	776	832	879	922		1006	1065	1115	1182	1226	1709*		β4
23 (NM_001362504.1)	12301	w (NP_001349433.1)	1707		59			86	154	226	267	325		*Mir204*	383		424	449	482	527	544		591	601	698	774	830	877	920		1004	1063	1113	1180	1224	1707*		β2
24 (NM_001362505.1)	12269	p (NP_001349434.1)	912											*Mir204*													35	82	125		209	268	318	385	429	912*		
25 (NM_001362506.1)	12447	y (NP_001349435.1)	1689		59			86	154	226	267	325		*Mir204*	383		424	449	482	509	526		573	583	680	756	812	859	902		986	1045	1095	1162	1206	1689*		β7
26 (NM_001362507.1)	12253	z (NP_001349436.1)	1691		59			86	154	226	267	325		*Mir204*	383		424	449	482	509	526	548	585		682	758	814	861	904		988	1047	1097	1164	1208	1691*		β9
27 (NM_001362508.1)	9526	aa (NP_001349437.1)	785											*Mir204*																	82	141	191	258	302	785*		
28 (NM_001362511.1)	2130	bb (NP_001349440.1)	262						1	73	114	172	197	*Mir204*	255	262*																						
29 (NM_001362512.1)	2701	cc (NP_001349441.1)	259						1	73	114	172	197	*Mir204*	255	259*																						
30 (NM_001362513.1)	3136	dd (NP_001349442.1)	265						1	73	114	172	197	*Mir204*	265*																							
B. Predicted (GRCm38.p6 C57BL/6J)																																						
X1 (XM_006526954.4)	13700	X1 (XP_006527017.1)	1758	61				88	156	228	269	327	352	*Mir204*	410		451	476	509	554	571	583	630	640	737	813	869	916	959		1043	1114	1164	1231	1275	1758*		
X2 (XM_006526955.4)	12473	X2 (XP_006527018.1)	1756		59			86	154	226	267	325	350	*Mir204*	408		449	474	507	552	569	581	628	638	735	811	867	914	957		1041	1112	1162	1229	1273	1756*		
X3 (XM_006526956.4)	13671	X3 (XP_006527019.1)	1748	61				88	156	228	269	327	352	*Mir204*	410		451	476	509	554	571	583	630		727	803	859	906	949		1033	1104	1154	1221	1265	1748*		
X4 (XM_006526957.4)	13663	X4 (XP_006527020.1)	1746	61				88	156	228	269	327	352	*Mir204*	410		451	476	509	554	571	583	630	640	737	813	869	916	959		1043	1102	1152	1219	1263	1746*		
X5 (XM_006526958.4)	13644	X5 (XP_006527021.1)	1746	61				88	156	228	269	327	352	*Mir204*	410		451	476	509	554	571		618	628	725	801	857	904	947		1031	1102	1152	1219	1263	1746*		
X6 (XM_017318155.2)	13634	X6 (XP_017173644.1)	1736	61				88	156	228	269	327	352	*Mir204*	410		451	476	509	554	571	583	630		727	803	859	906	959		1033	1092	1142	1209	1253	1736*		
X7 (XM_006526961.4)	13627	X7 (XP_006527024.1)	1734	61				88	156	228	269	327	352	*Mir204*	410		451	476	509	554	571		618	628	725	801	857	904	947		1031	1090	1140	1207	1251	1734*		
X8 (XM_006526963.4)	13625	X8 (XP_006527026.1)	1733	61				88	156	228	269	327		*Mir204*	385		426	451	484	529	546	558	605	615	712	788	844	891	934		1018	1089	1139	1206	1250	1733*		
X9 (XM_006526966.4)	13597	X9 (XP_006527029.1)	1724	61				88	156	228	269	327	352	*Mir204*	410		451	476	509	554	571		618		715	791	847	894	937		1021	1080	1130	1197	1241	1724*		
X10 (XM_006526974.4)	12296	X10 (XP_006527037.1)	1697		59			86	154	226	267	325		*Mir204*	383		424	449	482	527	544		591		688	764	820	867	910		994	1053	1103	1170	1214	1697*		
X11 (XM_006526975.3)	12538	X11 (XP_006527038.1)	1603						1	73	114	172	197	*Mir204*	255		296	321	354	399	416	428	475	485	582	658	714	761	804		888	959	1009	1076	1120	1603*		
X12 (XM_006526976.4)	12405	X11 (XP_006527039.1)	1603						1	73	114	172	197	*Mir204*	255		296	321	354	399	416	428	475	485	582	658	714	761	804		888	959	1009	1076	1120	1603*		
X13 (XM_006526978.3)	12462	X12 (XP_006527041.1)	1578						1	73	114	172		*Mir204*	230		271	296	329	374	391	403	450	460	557	633	689	736	779		863	934	984	1051	1095	1578*		
X14 (XM_006526979.4)	12044	X13 (XP_006527042.1)	1376	61				88	156	228	269	327	352	*Mir204*	410		451	476	509	554	571	583	630	640	737	813	869	916	959		1043	1114	1164	1231	1275	1370	1376*	
X15 (XM_011247233.3)	12008	X14 (XP_011245535.1)	1364	61				88	156	228	269	327	352	*Mir204*	410		451	476	509	554	571	583	630	640	737	813	869	916	959		1043	1102	1152	1219	1263	1358	1364*	
X18 (XM_030250887.1)	10484	X15 (XP_030106747.1)	785											*Mir204*																	82	141	191	258	302	785*		
X19 (XM_030250888.1)	6288	X16 (XP_030106748.1)	420	61				88	156	228	269	327	352	*Mir204*	420*																							
X20 (XM_030250889.1)	3952	X17 (XP_030106749.1)	414	61				88	156	228	269	327	352	*Mir204*	410	414*																						
X21 (XM_017318158.1)	3877	X18 (XP_017173647.1)	389	61				88	156	228	269	327		*Mir204*	385	389*																						
