# Supplementary material for: TRPM3_miR-204: a complex locus for eye development and disease
Source: Hum Genomics. 2020 Feb 18;14:7. doi: 10.1186/s40246-020-00258-4 (PMC7027284; doi:10.1186/s40246-020-00258-4)
Supplement: Supplementary file 3 — Additional file 3. Table S3. Ocular expression profile of the TRPM3 and miR-204 genes in humans (TRPM3, MIR204) and mice (Trpm3, Mir204) [file 40246_2020_258_MOESM3_ESM.pdf]

Supplementary Table 3. Ocular expression profile of TRPM3 and miR-204 genes in humans (*TRPM3* , *MIR204*) and mice (*Trpm3* , *Mir204*).

**Eye Tissue or Cell Type**	**EST/cDNA (neibank.nei.nih.gov)**	**Northern Blot**	**RT-PCR**	**qPCR**	**ISH**	**Microarray (biogps.org)**	**RNA-Seq**	**Reporter Transgene (β-gal)**	**Western Blot**	**IHC**	**Reference**
Whole eye (m)						*Trpm3*					**93**
Cornea (h/m), corneal epithelial cells		*Mir204*			*Mir204*	*Trpm3*	*MIR204*				**134, 156, 158**
Iris (h, m)	*TRPM3*					*Trpm3*					**129, 130**
Ciliary Body (h/m) non-pigmented epithelium					*Trpm3,* *Mir204* *MIR204*	*Trpm3*	*MIR204*	*Trpm3*			**93, 134, 139, 146, 147, 155, 156**
Trabecular meshwork (h), HTM cells						*MIR204*	*MIR204*				**134, 141-143**
Lens (h/m), lens epithelial cell-lines	*TRPM3*	*Mir204*	*TRPM3*		*Trpm3* *Mir204* *MIR204*	*Trpm3*	*Mir204* *Trpm3* *TRPM3*				**81, 129, 132, 137, 138, 139, 140, 146, 148, 149, 150, 156, 165**
Retina (h/m)	*TRPM3*	*Trpm3* *Mir204*	*Trpm3* *Mir204*		*Mir204* *MIR204*	*Trpm3*	*MIR204* *Mir204*				**93, 131, 133, 139, 146, 147, 151, 155-157, 159-163**
Eye/Optic Cup (m)		*Trpm3*				*Trpm3*					**147**
GCL (m), Muller glia cells			*Trpm3*		*Trpm3* *Mir204* *MIR204*			*Trpm3*		*Trpm3*	**83, 93, 146, 153, 163**
IPL (m) - outer OFF sublamina a										*Trpm3*	**153**
INL (h/m)					*Trpm3* *Mir204* *MIR204*					*Trpm3*	**83, 93, 146, 147, 151, 153**
OPL (m)										*Trpm3*	**153**
ONL (h) (rod/cone)					*MIR204*						**83**
RPE (h/m), ARPE-19 cells	*TRPM3*	*MIR204*		*TRPM3* *MIR204*	*Trpm3* *Mir204* *MIR204*	*Trpm3*	*MIR204* *TRPM3* *Mir204*		*TRPM3*	*TRPM3*	**83, 131, 133, 135-139, 144-146, 155**
Optic nerve head, optic nerve glia (m)			*Trpm3*	*Trpm3*						*Trpm3*	**152, 154**
